# In search of the *Aplysia* immunome: an in silico study

**DOI:** 10.1186/s12864-022-08780-6

**Published:** 2022-07-29

**Authors:** Nicholas S. Kron

**Affiliations:** Department of Marine Biology and Ecology, Rosenstiel School of Marine and Atmospheric Science, University of Miami, 4600 Rickenbacker Cswy, Miami, FL 33149 USA

**Keywords:** Innate immunity, Toll-like receptor, RIG-I-like receptor, MAVS, STING (min. 3-max. 10)

## Abstract

The immune repertoires of mollusks beyond commercially important organisms such as the pacific oyster *Crassostrea gigas* or vectors for human pathogens like the bloodfluke planorb *Biomphalaria glabrata* are understudied. Despite being an important model for neural aging and the role of inflammation in neuropathic pain, the immune repertoire of *Aplysia californica* is poorly understood. Recent discovery of a neurotropic nidovirus in *Aplysia* has highlighted the need for a better understanding of the *Aplysia* immunome. To address this gap in the literature, the *Aplysia* reference genome was mined using InterProScan and OrthoFinder for putative immune genes. The *Aplysia* genome encodes orthologs of all critical components of the classical Toll-like receptor (TLR) signaling pathway. The presence of many more TLRs and TLR associated adapters than known from vertebrates suggest yet uncharacterized, novel TLR associated signaling pathways. *Aplysia* also retains many nucleotide receptors and antiviral effectors known to play a key role in viral defense in vertebrates. However, the absence of key antiviral signaling adapters MAVS and STING in the *Aplysia* genome suggests divergence from vertebrates and bivalves in these pathways. The resulting immune gene set of this in silico study provides a basis for interpretation of future immune studies in this important model organism.

**Supplementary Information:**

The online version contains supplementary material available at 10.1186/s12864-022-08780-6.

## Background

Making inferences of immune function from genomics and transcriptomics data can be challenging with non-mammalian or non-ecdysozoan models due to a paucity of functional research and gene characterization outside these two groups. Immune research in mollusks has historically centered on commercially important species such as the Pacific oyster *Crassostrea gigas* [1–3], or on models of parasite transmission such as the bloodfluke planorb *Biomphalaria glabrata* [4–6]. Recent work has ventured to describe immune function in other mollusks, mainly in other commercially important bivalves [7–9], but also in a few important molluscan models such as the pond snail *Lymnaea stagnalis* [10] and the common octopus *Octopus vulgaris* [11]. These organisms often lack reference genomes or transcriptomes, and researchers must rely on de novo assembly and homology-based transcript annotation. Many other mollusk models for which publicly available reference sequences are available have not been comprehensively surveyed for immune related genes, such as the marine model *Aplysia californica*.

Despite being a premier neural model for more than half a century and boasting a reference genome and transcriptome, the immune repertoire of *Aplysia* is still poorly understood [12–14]. Historically, the extent of interest in the *Aplysia* immune response was a model for the role of inflammation and immune response in neuropathic pain [15–18]. Recently, however, a novel virus in the order Nidovirales was discovered in the marine model *Aplysia californica* [19]. This virus appears to have a broad tropism, with particularly high viral expression levels in the nervous system [20]. This discovery presents an opportunity to further develop *Aplysia* as a new model for neurotropic viral infection, particularly in the context of neural aging. However, the dearth of functional annotation and lack of even a putative immune gene set makes future inference into the immune response to this novel virus difficult. While some putative immune associated genes have been identified in *Aplysia* previously in the context of the neuronal transcriptome [14] or aging associated neuro-inflammation [21], these studies did not perform a comprehensive survey of the *Aplysia* immunome. To address this gap in the *Aplysia* literature, an in silico survey of *Aplysia* reference genome was conducted to identify and describe a putative immunome to aid in future immunological studies in this well-established model.

## Results

A combination of InterProScan (IPS) protein domain annotation and OrthoFinder gene homology search hierarchical clustering was used to identify genes in the *Aplysia* genome with potential for immune function (see Methods). Candidate immune genes were first identified by conserved protein domains typical of immune genes in other taxa and then vetted for ortholoy to said immune genes with OrthoFinder. This was particularly necessary for genes which cannot be easily identified by diagnostic conserved domains alone, such as inhibitors of NFkB. Of the 26,658 predicted proteins in the AplCal3.0 gene feature format file (gff) available from the NCBI RefSeq FTP site, 26,209 (98.3%) were annotated with at least one analyses incorporated into IPS, and 22,511 (84.4%) proteins were annotated with at least one InterPro accession.

From the 5 species used for OrthoFinder analysis (Human, *Drosophila melanogaster*, *Crassostrea gigas*, *Biomphalaria glabrata*, and *Aplysia californica*), proteins were sorted into 19,353 orthogroups. Of these orthogroups, 12,594 (65.1%) contain genes from at least 2 species while 4606 (23.8%) contained genes from across all 5 species surveyed. Comparing among groups, 1,723 orthogroups were common to mollusks and humans but not the ecdysozoan *Drosophila*. A further 1501 orthogroups were common only to the mollusks surveyed and 1918 were common only to the two gastropods. A total of 849 orthogroups were unique to *Aplysia*.

By mining the IPS results for immune domain signatures (see Methods) and vetting the resultant set with OrthoFinder results, 2241 *Aplysia* proteins from 1669 genes were identified as having potential immune function. These putative immune genes are described in detail below.

IPS annotation of the *Aplysia californica* RefSeq predicted protein models can be found in supplemental file SFile1. Full OrthFinder results can be found in supplemental file SFile2. The fully compiled immunome can be found in supplemental file SFile3.

### Pattern recognition receptors (PRRs)

#### Glycan receptors

Several PRRs are adapted to detect the glycans of various pathogens, including peptidogylcans, beta 1,3-glucan from fungi, and lipoglycans like bacterial liposaccharide (LPS) [22–24]. Many such PRRs were identified in *Aplysia*, including: five proteins in the CD36-like family, three gram-negative bacteria binding-proteins (LOC101850763, LOC101851233, LOC101861522), and two peptidoglycan recognition proteins (LOC101855568, LOC101858995). Orthologs of Beta 1,3-glucan recognition proteins (BGRP) which are antimicrobial PRRs known from insects, were not identified, however. In vertebrates, detection of bacterial liposaccharides (LPS) is facilitated by an extracellular cascade of the LPS binding proteins LBP, CD14, and MD2. LPB binds LPS and facilitates its binding to CD14, which then delivers LPS to the MD-TLR4 receptor complex [25]. A single LBP protein, LOC101845376, as well as 15 proteins with MD-2-related lipid-recognition domains were identified, however potential orthologs of CD14 were not (Table 1).

**Table 1:** Putative glycan receptor genes identified in the *Aplysia*. Conserved protein domains identified by InterProScan used to identify each immune gene set are listed under “Required Domains.” The Strict column contains diagnostic domains know from vertebrate orthologs of target genes. The relaxed column represents more relaxed criteria, using family level annotation for domains that are more divergent in invertebrate lineages. The count column contains the number of genes identified for each group based on mining IPS results for requisite domains and then vetting with OrthoFinder predicted orthologs. Genes with fewer than 5 representatives per group have their gene identifiers listed, otherwise readers are encouraged to access Supplemental File SFile3 for full gene and protein identifier lists

	Required Domains		Genes	
Name	Strict	Relaxed	Count	Accessions
CD36-like	IPR005428	IPR002159	5	See supplemental file SFile3
GNBP	IPR000757		3	LOC101861522, LOC101850763, LOC101851233
PGRP	IPR006619		2	LOC101858995, LOC101855568
MD2	IPR003172	IPR039217	15	See supplemental file SFile3
LBP/BPI	IPR001124, IPR017942	IPR030675	1	LOC101845376

#### Lectins

Lectins are a diverse group of multifunctional proteins defined by their carbohydrate recognition domains (CRD). Many lectins have been implicated in immune sensing of pathogen associated molecular patterns (PAMPs) across taxa, particularly C-type lectins [26]. The *Aplysia* genome encodes a diverse collection of lectins including: 15 Apextrins, 146 C-type lectin domain containing proteins (CTLD), 33 Chi-type lectins, 5 F-type lectins, 96 fibrinogen domain containing proteins (FBGDC), 5 galectins, 5 H-type lectins, 100 i-set immunoglobulin proteins which may contain i-type lectins, 2 L-type lectins, 6 M-type lectins, 13 P-type lectins, 4 pentraxins, 21 R-type lectins, and 3 SUEL lectins were identified (Table 2). No mannose-binding lectins or f-box lectins were identified.

**Table 2:** Putative lectin genes identified in *Aplysia*. See Table 1 for table format description

	Required Domains		Genes	
Name	Strict	Relaxed	Count	Accessions
Apextrin	IPR031569		15	See supplemental file SFile3
Chi-lectin	IPR001223		33	See supplemental file SFile3
CREP	IPR001304, IPR007110	IPR001304, IPR036179	2	LOC101860578, LOC101862063
CTLD	IPR001304	IPR016187	146	See supplemental file SFile3
F-type lectins	IPR006585		5	LOC101861054, LOC101863833, LOC106011541, LOC106012254, LOC118477646
FBGDC	IPR002181, IPR007110	IPR001304, IPR036179	96	See supplemental file SFile3
FREP*	IPR002181		2	LOC100533300, LOC100533436
Galectin	IPR001079		5	See supplemental file SFile3
H-type lectin	IPR019019	IPR037221	5	LOC101855237, LOC101859099, LOC101863352, LOC101862976, LOC118477875
I-type lectins	IPR013098		100	See supplemental file SFile3
L-type lectin	IPR005052		2	LOC101854301, LOC106011450
M-type lectin	IPR001382		6	See supplemental file SFile3
P-type lectin	IPR044865	IPR009011	13	See supplemental file SFile3
Pentraxins	IPR030476	IPR001759	4	See supplemental file SFile3
R-type lectins	IPR000772		21	See supplemental file SFile3
SUEL lectin	IPR000922		3	LOC101858932, LOC101863218, LOC101864702

A unique subclass of lectins that pairs an immunoglobulin superfamily (IgSf) domain with a carbohydrate recognition domain, called Variable Immunoglobulin and Lectin domain containing molecules (VIgL), have been shown to play an important role in innate immunity in gastropods [4]. The first identified VIgL in *Biomphalaria* was dubbed a fibrinogen related protein (FREP). Later, genes with similar domain architecture but galectin or C-type lectin carbohydrate recognitions domains were dubbed GREP and CREP respectively.

**Fig. 1:**
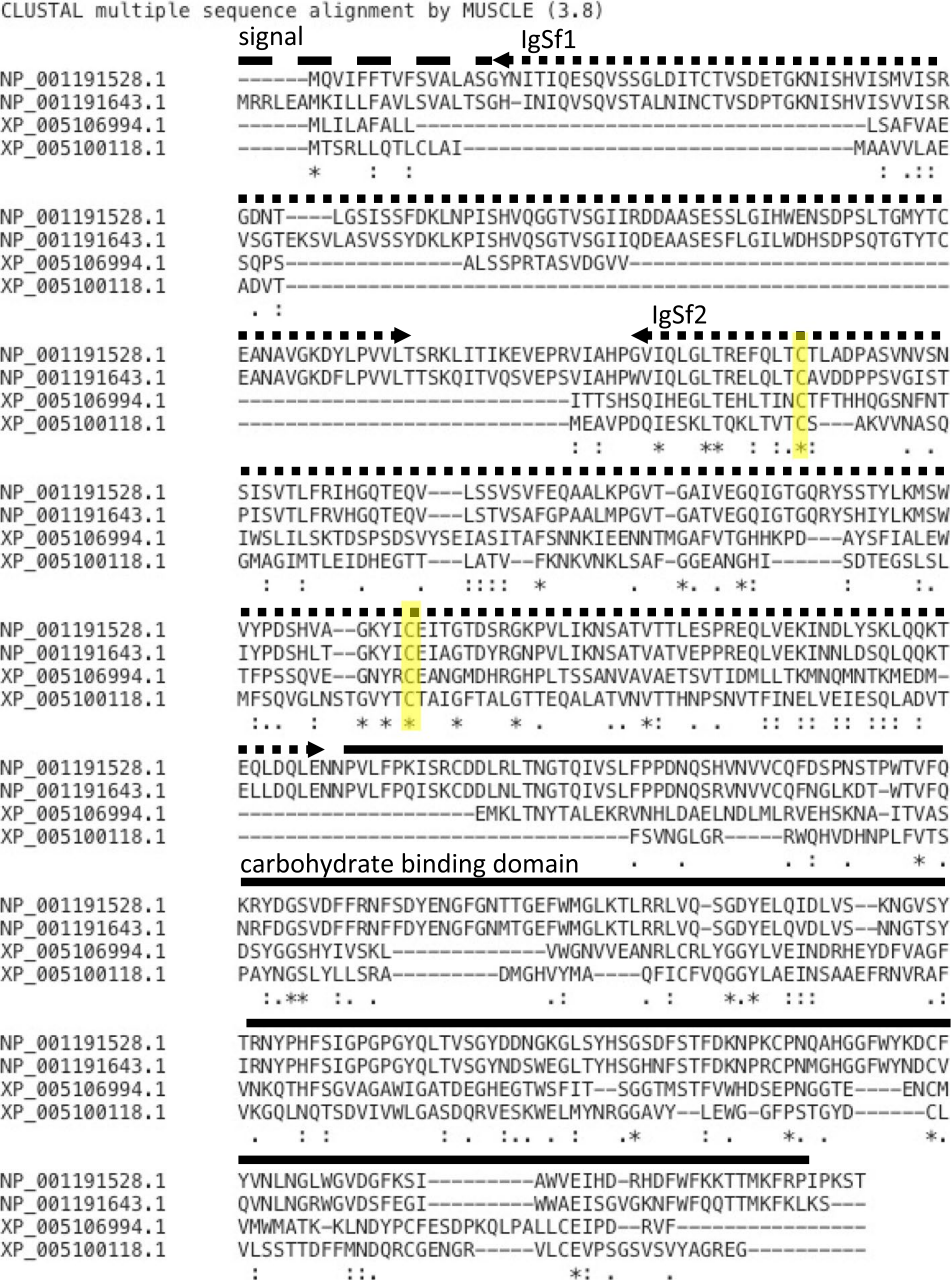
Conserved domains of Variable Immunoglobulin-like (VIgL) genes in *Aplysia californica*. Multiple sequence alignment of two previously identified *Aplysia* FREP proteins (NP_001191528/LOC100533300 and NP_001191643/LOC100533436) and two putative CREP proteins (XP_005106994/ LOC101862063 and XP_005100118/ LOC101860578) identified by InterProScan conserved domains. Above the alignment, a set of lines demarcate the protein domains of the two previously described *Aplysia* FREPs as reported in [27]. Dashed line above sequence alignments demarcates signal peptide. Dotted line demarcates immunoglobulin superfamily (IgSf) domains. Solid line demarcates the carbohydrate binding domains. Conserved cysteine residues of VIgLs IgSf domains are highlighted in yellow. Note that the putative CREPs only contain one IgSf domain and thus the signal peptides alignments extend into the region demarcated as IgSF1 for the FREPs

Based on conserved domains identified in IPS, 2 possible CREP genes (LOC101860578 and LOC101862063) were identified. It was not possible to find any FREPs based on conserved domains, although two Aplysia FREPs have been reported previously. Because IgSf domains of mollusk VIgLs are difficult to detect for automated software such as those used by InterProScan [10], BLAST homology searches to known gastropod VIgLs from *Biomphalaria* were used to identify these subsets of FBGDC, CTLDC, and galectin domain containing genes. This approach recovered the two previously identified FREPs [27] and aforementioned CREPs. Multiple sequence alignment of the two putative CREPs with previously described Aplysia FREPs via Clustal Omega identified potentially distantly related IgSf regions (Fig. 1) [28].

#### Nucleotide receptors

**Fig. 2:**
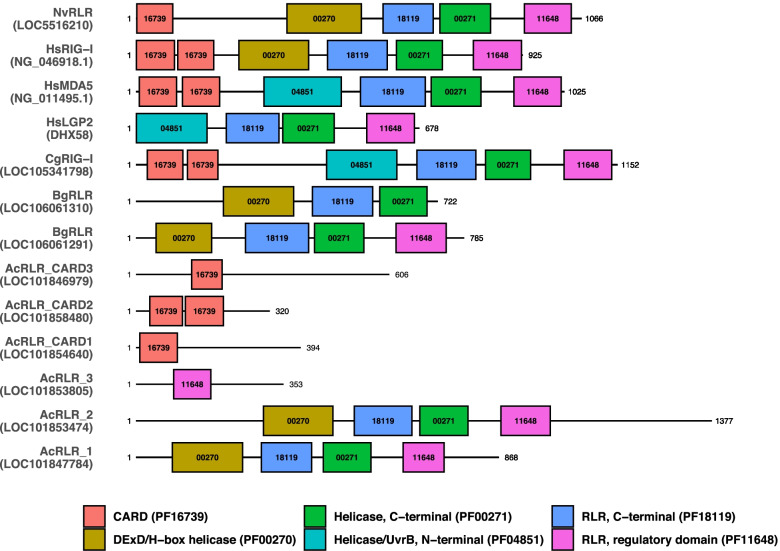
Conserved domains of RIG-I-like receptors in Aplysia and other animals. Proteins are represented by black line segments and labeled according to species with gene identifiers provided in parentheses (Nv = *Nematostella vectensis,* Hs *= Homo sapiens,* Cg *= Crassostraea gigas,* Bg *= Biomphalaria glabrata,* Ac *= Aplysia californica*). Colored boxes along gene line segments represent conserved protein domains identified by InterProScan. Numbers inside boxes representing conserved domains are Pfam identifiers with “PF” trimmed off (e.g. PF00270 ➔ 00270)

To detect infection by pathogens, animals employ a diverse array of nucleotide sensing PRRs to alert intracellular immune cascades to the presence of pathogenic DNA or RNA in the cytosol. The quintessential cytosolic PRR sensor is RIG-I, which detects viral RNAs [29]. Three proteins with RIG-I-like c-terminal domains (CTD) involved in viral RNA recognition were identified. Two of these, LOC101847784 and LOC101853474, contain DExD/H-box helicase domains in addition to RIG-I-like CTDs, whereas LOC101853805 lacked any identifiable domain other than the RIG-I-like CTD. As in *Biomphalaria*, all three proteins lacked CARD domains typical of vertebrate and bivalve RLRs (Fig. 2). Two additional genes were identified as RLRs by OrthoFinder homology search (LOC101858480 and LOC101846979). These genes contain RIG-I/MAVS-type CARD (IPR031964) domains but lack any DExD/H-box helicase domains or RLR CTD. One of these, labeled as an IFIH1/MDA5 ortholog in the REFSeq database (LOC101858480), contains two CARD domains typical of vertebrate/bivalve RIG-I and MDA. Although RIG-I acts to detect viral RNAs, RNA Polymerase III can allow RIG-I to detect DNA viruses as well by converting AT-rich viral DNAs into RNAs detectable by RIG-I [29]. The RPC34-like subunit of the RNA Polymerase III was identified: LOC101860996.

In addition to RLRs, several other DExD/H-box helicases have been implicated in immune sensing [30]. Fifty-nine other DExD/H-box helicase genes were identified in *Aplysia*. Among these are specific DExD/H-box helicases known to play a role in the immune response of vertebrates, including DDX1 (LOC101857175), DDX41(LOC101864496), DHX9 (LOC101848799), and DHX15 (LOC101860724). Furthermore, the *Aplysia* genome contains 12 DEAD-box OB fold, also called domain of unknown function 1605, containing DExD/H-box helicases. This group contains other known vertebrate immune-associated genes including DHX29/30/33/36 and the aforementioned DHX15. Similarly, the *Aplysia* genome also contains 26 Q-motif containing DExD/H-box helicases, a group that contains still other known vertebrate immune-associated genes including DDX 3/6/17/19/23/24/39/46/56 in addition to aforementioned DDX1 and DDX41.

In parallel to RIG-I and DExD/H-box helicases, zinc finger NFX1-type containing 1 (ZNFX1), has also been implicated as a dsRNA sensor and antiviral protein in metazoans [31]. Using conserved domains, PANTHER annotation from IPS (PTHR10887:SF470), and homology search, three potential ZNFX1 genes (LOC101862822, LOC101857249, LOC101851452) were identified.

Several DNA-damage detecting proteins have also been implicated in cytosolic sensing of viral nucleotide [32, 33]. Putative orthologs of several of these, including all three subunits of the DNAPK complex [34] (DNAPKc LOC101854982, Ku70 LOC101860888, and Ku80 LOC101855590); both components of the MRN complex [35, 36] (MRE11 LOC101858081 and RAD50 LOC101847050); and several Mab-21 domain containing proteins that may be orthologs of vertebrate cGAS (LOC101848176, LOC101852616, LOC101863483) were identified. In addition to DNA-damage associated sensors, orthologs to several other potential cytosolic viral sensors were identified, including LRRFIP1 (LOC101862386) and its downstream signaling partner β-catenin (LOC100533383); and 32 High Mobility Group Box proteins (Table 3).

**Table 3:** Putative nucleotide receptor genes identified in *Aplysia*. See Table 1 for table format description

	Required Domains		Genes	
Name	Strict	Relaxed	Count	Accessions
ABCF1	IPR003593, IPR003439		83	See supplemental file SFile3
cGAS	IPR024810		3	LOC101848176, LOC101852616, LOC101863483
DNAPK	IPR037706, IPR045581, IPR012582	IPR037706	1	LOC101854982
HMGBs	IPR009071	IPR036910	32	See supplemental file SFile3
Ku70/80	IPR006164, IPR005160, IPR005161		2	LOC101860888, LOC101855590
LRRFIP1	IPR019139		1	LOC101862386
MRE11	IPR041796, IPR007281	IPR038487, IPR004843	1	LOC101858081
NLR	IPR007111, IPR001611	IPR007111, IPR032675	1	LOC101849611
RAD50	IPR038729, IPR045171, IPR013134	IPR027417, IPR004584	1	LOC101847050
RLR	IPR011545, IPR041204, IPR021673	IPR038557	3	LOC101853474, LOC101853805, LOC101847784
RNApol III	IPR007832, IPR036390	IPR016049, IPR036388	1	LOC101860996

**Fig. 3:**
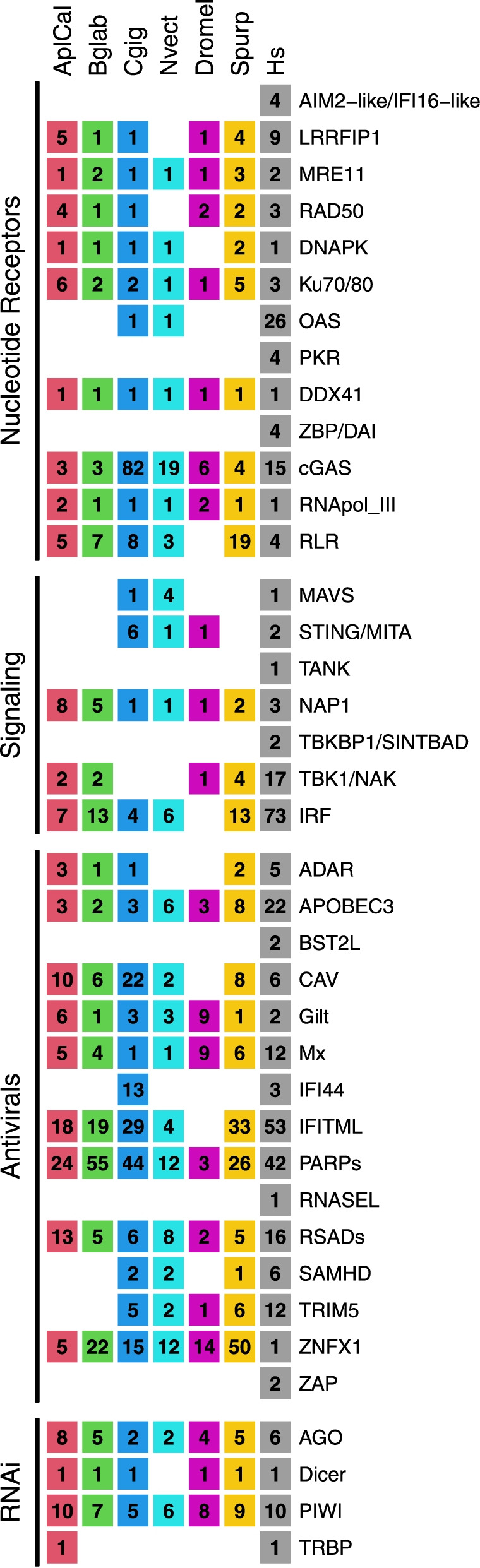
Orthologous components of the antiviral signaling cascade in *Aplysia californica* and other animals. Immune genes of interest were extracted from a custom InterProScan annotation of the *Aplysia californcia* RefSeq protein modles (AplCal, GCF_000002075.1) and publicly available InterPro annotations of proteins from the UniprotKB data base for *Biomphalaria glabrata* (Bglab, UP000076420_IPS), *Crassosteraea gigas* (Cgig, UP000005408), *Nematostella vectensis* (Nvect, UP000001593), *Drosophila melanogaster* (Dromel, UP000000803), *Strongylocentrotus purpuratus* (Spurp, UP000007110)*,* and human (Hs, UP000005640) based on InterPro domains detailed in supplementary data Supplemental File SFile5. Results were further refined using sequence similarity search among listed proteomes and predicted protein models using OrthoFinder and BLASTP. Columns of the table represent a single species, while rows represent proteins known to play key roles in antiviral signaling in mammals. Proteins are grouped according to their functional roles in antiviral signaling: viral nucleotide receptors, signaling cascade components such as adapters like STING and MAVS, antiviral effector genes such as ADAR, and components of RNA interference (RNAi) known to play a major role in arthropod viral response. Numbers in each cell represent the number of protein hits to each protein type, and thus differ from gene level numbers present in the main text. Note that hits are to Uniprot KB proteomes and as such differ from previously reported numbers for *C. gigas* and *S. purpuratus* which used predicted gene models. While *Aplysia* retains many viral sensors and antiviral effectors, key signaling adapters STING and MAVS are absent despite being retained in Oyster. Pyrin and hematopoietic interferon-inducible nuclear (HIN) domain (PYHIN) proteins (AIM2-like/IFI16-like); Leucine-rich repeat flightless-interacting protein 1-like (LRRFIP1); Double-strand break repair protein MRE11-like (MRE11); DNA repair protein RAD50 (RAD50); DNA-dependent protein kinase-like (DNAPK); ATP-dependent DNA helicase II/X-ray repair cross-complementing protein 5/6 (Ku70/80); 2′-5′-oligoadenylate synthetase (OAS); High mobility group box proteins (HMGBs); EIF2AK2/PKR-like (PKR); ATP-dependent RNA helicase DDX41 (DDX41); Z-DNA-binding proteins (ZBP/DAI); Cyclic GMP-AMP synthase (cGAS); DNA-directed RNA polymerase III (RNApol_III); RIG-I-like receptors (RLR); Mitochondrial Antiviral-Signaling Protein (MAVS); Stimulator of interferon genes (STING/MITA); TRAF family member-associated NF-kappa-B activator (TANK); Nck-associated_protein-1 (NAP1); TANK-binding kinase 1-binding protein 1 (TBKBP1/SINTBAD); TANK-binding kinase 1/ NFkB activated Kinase (TBK1/NAK); Interferon regulatory factor (IRF); Double-stranded RNA-specific adenosine deaminase (ADAR); Apolipoprotein B Editing Complex 3 proteins (APOBEC3); Tetherines (Bone marrow stromal antigen 2-like) (BST2L); Caveolin (CAV); Gilt (Gilt); Interferon-induced GTP-binding protein Mx (Mx); Interferon-induced protein 44 (IFI44); Interferon-induced transmembrane protein-like (IFITML); Protein mono-ADP-ribosyltransferase (PARPs); Ribonuclease L (RNASEL); viperins (Radical S-adenosyl methionine domain-containing proteins) (RSADs); Deoxynucleoside triphosphate triphosphohydrolase SAMHD (SAMHD); Tripartite motif-containing protein 5 (TRIM5); zinc finger NFX1-type containing 1 (ZNFX1); Zinc-finger antiviral proteins (ZAP); Argonaute (AGO); Dicer (Dicer); Piwi-like (PIWI); RISC-loading complex subunit TARBP (TRBP)

Notably, no putative orthologs of DNA-damage sensors DAI/ZBP, Pyrin and hematopoietic interferon-inducible nuclear (HIN) domain (PYHIN) proteins [34], or viral RNA sensors inducible oligoadenylate synthetase (OAS) and Protein Kinase R [37, 38] were identified (Fig. 3).

#### Broad Spectrum PRR families

While several families of PRRs are dedicated to a specific class of PAMPs, the Toll-like receptor family of PRRs can detect a diverse range of PAMPs. For example, in mammals TLRs can detect bacterial lipopeptides (TLR1,2,3), glycans (TLR4), nucleic acids (3,7,8,9), and bacterial proteins (TLR2,5) [39]. In Aplysia 39 genes with Toll/interleukin-1 receptor homology (TIR) domain and Leucine Rich Repeat (LRR) domains characteristic of TLRs were identified. OrthoFinder divided these putative TLRs in 17 orthogroups. Of these, four orthogroups were unique to *Aplysia* (OG0001270, OG0009332, OG0016038, and OG0009441). Of the 17, only three were not unique to gastropods, with OG0011109 and OG0012371 containing a protein from *C. gigas*, and OG0003873 containing proteins from *C. gigas* and *Drosophila*.

**Fig. 4:**
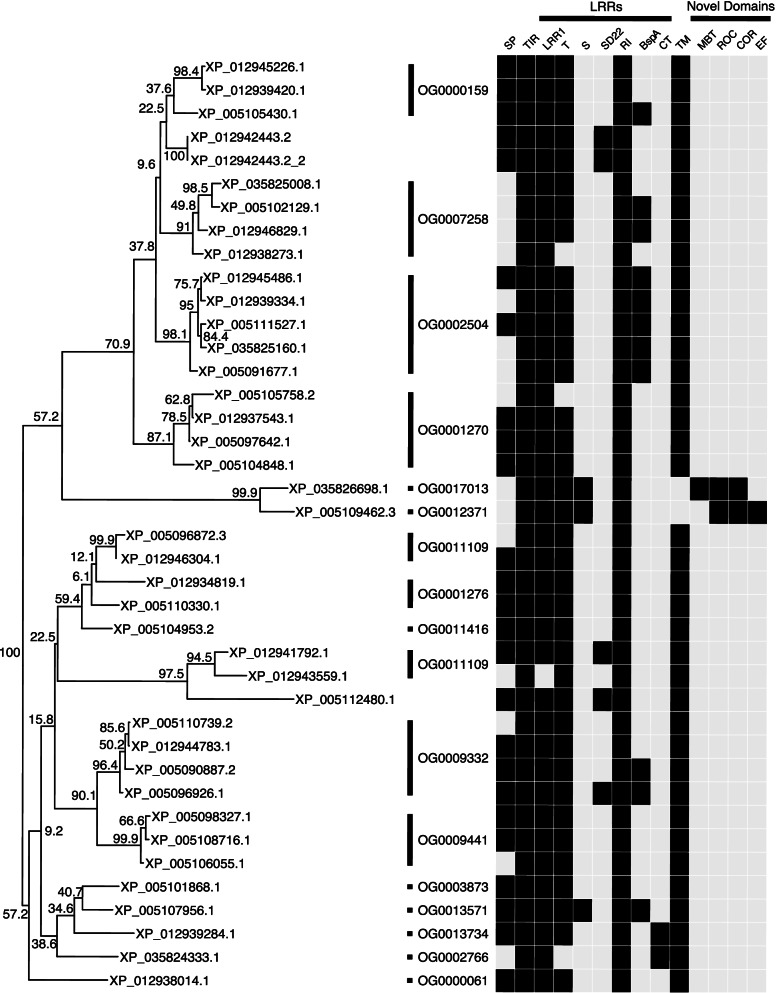
Neighbor Joining Tree of putative *Aplysia* Toll-like Receptor TLR domains. Neighbor joining tree generated using the Jones-Taylor-Thornton model and bootstrapped with 1000 iterations (see Methods) using the TIR domains of the longest protein isoform of all putative *Aplysia* TLR genes. Corresponding orthogroups identified in OrthoFinder analysis demarcate member proteins with strips and orthogroup identifier. Heatmap to the right of the tree represents presences (black square) or absence (light grey square) of key protein domains identified by InterProScan. Domain category is demarcated with a strip above the heatmap columns. Domains without a category include SP (signal peptide, Phobius), TIR (Toll-interleukin Receptor homology domain, IPR000157), TM (transmembrane domain, Phobius). Domains demarcated as LRRs represent various leucine-rich repeat subtypes: LRR1 (single LRR, IPR001611), T (typical type, IPR003591, SM00369), S (bacterial type, SM00364), SD22 (sds22-like, SM00365), BspA (BspA type, IPR026906), RI (Ribonuclease inhibitor, G3DSA:3.80.10.10), and CT (Cysteine-rich c-terminal flanking region of LRR, IPR000483, SM00082). Domains demarcated as “Novel Domains” represent domains found in Aplysia TLRs not typically associated with vertebrate TLRS, including MBT (malignant brain tumor repeat, IPR004092), ROC (Ras of complex small GTPase domain, IPR020859), COR (C-terminal of ROC, IPR032171), and EF (EF-hand, IPR002048). Only the TIR domains of XP_012942443.2 and XP_005112480.1 do not cluster with their Orthogroup. The TLRS contain Roc and Cor (ROCO) domains as well as other accessory domains, suggesting possible novel function. Only two TLRs contain c-terminal flanking regions

Neighbor joining tree with 1000 boostraps of TIR domain region of these putative TLRs broadly supports OrthoFinder’s division of TLR groups (Fig. 4). Only TIR domains from LOC101851910 (XP_005112480.1) and LOC101863390 (XP_012942443.2) clustered away from their assigned orthogroups (OG0001276 and OG0007258 respectively). However, these divergent TIR assignments corresponded to weekly supported branches with low bootstrap values.

Interestingly, LOC101863390 contains 2 TIR domains. In addition, LOC101863390 also contains 3 transmembrane domains, and two sets of LRRs that give the appearance of an end-to-end fusion of two TLRs.

**Fig. 5:**
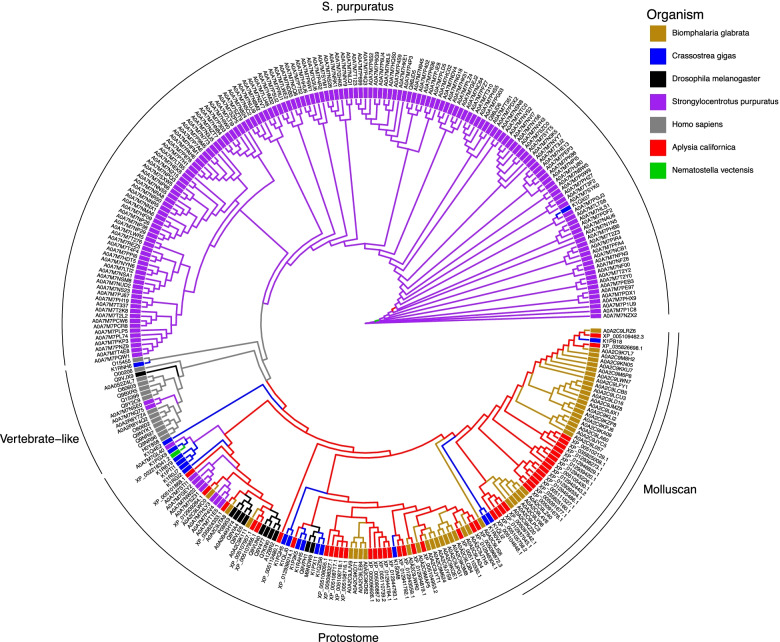
Multi-species Neighbor Joining Tree of Toll-like Receptor proteins. Toll-like receptor genes were extracted a custom InterProScan annotation of the *Aplysia californcia* RefSeq protein modles (AplCal, GCF_000002075.1) and publicly available InterPro annotations of proteins from the UniprotKB data base for *Biomphalaria glabrata* (Bglab, UP000076420_IPS), *Crassosteraea gigas* (Cgig, UP000005408), *Nematostella vectensis* (Nvect, UP000001593), *Drosophila melanogaster* (Dromel, UP000000803), *Strongylocentrotus purpuratus* (Spurp, UP000007110)*,* and human (Hs, UP000005640) based on presence of TIR and LRR domains. Protein sequences were aligned and used to generate a neighbor joining tree with the JTT model. Branches and leaf nodes are colored based on the species from which the sequence originated. Arcs describe major groupings of TLRs in the tree: a *S. purpuratus* dominated branch, a set of TLRs containing mostly human TLRs (dubbed vertebrate-like), a major branch that contains sequences only from the protostomes assessed (as well as lone TLR from *Nematostella*), and a branch containing only molluscan representatives. This tree corroborates earlier findings that massive TLR expansions in *S. purpuratus* and mollusks represent distinct, lineage specific radiations of TLR genes

Phylogenetic clustering of TLR sequences from *Aplysia californica*, *Biomphalaria glabrata*, *Crassosteraea gigas, Nematostella*, *Drosophila melanogaster*, *Strongylocentrotus purpuratus*, and humans revealed lineage specific trends in TLR diversification (Fig. 5). Molluscan TLRs clustered together with other protostome TLRs and exhibited a unique molluscan radiation within this group. This protostome radiation clustered apart from a massive radiation in *S. purpuratus*. The number of publicly available reference TLR protein sequences for *C. gigas* was substantially less than the manually annotated number previously reported by Zhang et al. (18 vs. 83) [3], making direct comparison difficult. However, these results do suggest a unique molluscan TLR radiation. Furthermore, TLRs from then two gastropods primarily originate from this uniquely molluscan radiation or its sister branch, unlike available *C. gigas* TLRs.

**Fig. 6:**
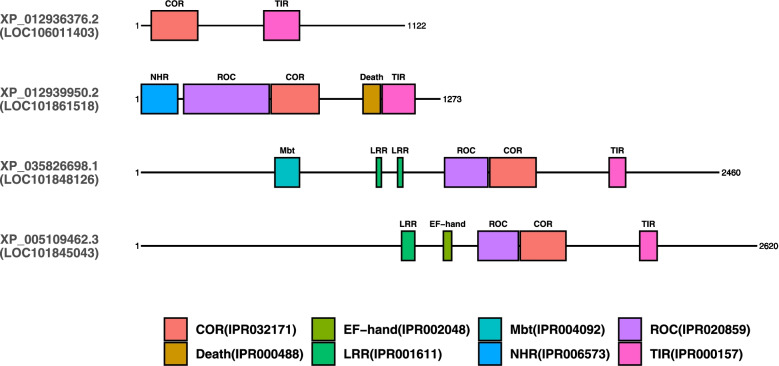
Conserved domains of TIR and COR domain containing proteins in *Aplysia*. Proteins are represented by black line segments labeled with RefSeq protein identifiers and gene locus number underneath in parentheses. Colored boxes along gene line segments represent conserved protein domains identified by InterProScan. Domains are labeled above (COR = C-terminal of Roc domain, Death = Death domain, EF-hand = EF-hand calcium-binding domain, LRR = Leucine-rich repeat, Mbt = Mbt repeat, NHR Neuralized homology repeat domain, ROC = Roc domain, TIR = Toll/interleukin-1 receptor homology domain)

While most of the *Aplysia* TLRs contain only TIR and LRR domains, several contain additional domains. Two of these putative TLRs (LOC101850809) and LOC101845154) contain detectable Cysteine-rich c-terminal flanking region in proximity of their LRR (Fig. 4). Another two putative TLRs contain the ROC and COR domains typical of ROCO GTPases. One (LOC101848126) contains an accessory Mbt repeat region while the other (LOC101845043) contains an EF-hand domain (Fig. 6). While these two genes are assigned to different orthogorups, tree analysis demonstrates they form a clade. Only two TLRs were identified as having c-terminal flanking regions (LOC101850809 and LOC101845154).

Like TLRs, Nucleotide-binding oligomerization domain-like receptors (NOD-like receptors, NLRs) are a diverse family of PRRs that detect a similarly broad range of PAMPs in vertebrates [40]. In *Aplysia*, only 4 proteins with the characteristic NACHT domain of NLRs were identified, only one of which contained both a NACHT domain and LRR domains typical of NLRs. This NLR-like protein also contains a Death-like domain and a DZIP3-like HEPN domain.

Like TLRs and NLRs, Receptor Cysteine-Rich (SRCR) Domains have been implicated in innate immune function across taxa against a broad suite of pathogens [41]. SRCR PRRs have been described in bivalves where they are found on the surface of haemocytes and respond to PAMPS [42]. In *Aplysia*, 11 SRCR genes were identified (Table 4).

**Table 4:** Putative broad spectrum pattern recognition receptor genes identified in *Aplysia*. See Table 1 for table format description

	Required Domains		Genes	
Name	Strict	Relaxed	Count	accessions
NLR	IPR007111, IPR001611	IPR007111, IPR032675	1	LOC101849611
SRCR	IPR001190		11	See supplemental file SFile3
TLR	IPR000157, IPR001611	IPR000157, IPR032675	39	See supplemental file SFile3

### Signaling

#### Adapter proteins

PRRs in several signaling cascades associate with adaptor proteins to facilitate transduction of pattern recognition into activation of kinases and ultimately transcriptional changes to facilitate an immune response, most notably the TLRs. Adaptor proteins of TLRs complex with their target receptor via their TIR domains. The most conserved TLR adaptor among animals is Myeloid differentiation primary response 88 (MyD88), which complexes with TLRs via its TIR domain and with Interleukin-1 receptor-associated kinases (IRAKs) via its Death-like domain (DLD) in a complex called a Myddosome [43]. In Aplysia 5 MyD88-like proteins were identified. One of these contained only the TIR and DLD typical of vertebrate MyD88 (LOC101855709), while three contained an additional Armadillo-type fold (LOC101850562, LOC101850562, and LOC101858605), and the last contained the same ROCO domain as described earlier in some AcTLRs (LOC101861518). OrthoFinder homology search also identified LOC101847182 as a MyD88-like protein based on TIR domain homology, but this gene lacked any death-like domains. In addition to MyD88, 4 other TIR domain containing (TIRDC) adaptors have been identified: MyD88-adaptor-like (Mal), TICAM1 and 2 (also known as TRIF and TRAM), and sterile-alpha and Armadillo motif protein (SARM) [44]. Although orthologs of MAL, TRIF, or TRAM could not be identified, three *Aplysia* SARMs (LOC101849069, LOC101850713, and LOC106011731) were identified. In addition, a diverse group of TIRDC proteins with as of yet unknown function were also identified. Of these, 10 contained Armadillo-type folds, two contained EGF-like domains, one contained COR domain, and the remaining 17 contained only a TIR domain. Potential orthologs of other, non-TIRDC TLR adaptor proteins Toll-Interacting Protein (TOLLIP, LOC101845996) and evolutionarily conserved signaling intermediate in Toll pathway (ECSIT, LOC101857763) were also identified.

One of the several downstream kinase complexes associated with PRR activation is the TAK1-TAB complex. The TAB proteins TAB1-3 facilitate the association of the kinase TAK1 with ubiquitin chain scaffolds to colocalize with downstream targets [45]. A TAB1 ortholog was difficult to identify based on protein domain alone, as the only InterPro domain in vertebrate TAB1 is the PPM-type phosphatase domain (IPR001932) which is found in 14 Aplysia genes. However, OrthoFinder homology search identified a gene with two isoforms (LOC101854409) listed as TAB1 on the NCBI. The other TAK1 adaptors TAB2 and 3 were similarly difficult to identify based on IPS alone. It was not possible to identify vertebrate-like TAB2/3 proteins that contain both a CUE domain and RAnBP2 zinc finger. OrthoFinder homology search identified two isoforms of the same gene (LOC101854409), which is identified as TAB2 on the NCBI. Notably, this gene lacks any CUE domain but does contain a RAnBP2 zinc finger needed for ubiquitin chain binding and subsequent signaling [46]. In fact, 19 RanBP2-type zinc finger domain containing genes were identified (Table 5).

**Table 5:** Putative immune signaling adapter genes identified in *Aplysia*. See Table 1 for table format description

	Required Domains		Genes	
Name	Strict	Relaxed	Count	Accessions
ARM-TIR	IPR000157, IPR000225	IPR035897, IPR016024	11	See supplemental file SFile3
ECSIT	IPR029342	IPR010418	1	LOC101857763
EGF-TIR	IPR000157, IPR000742		2	LOC101855416, LOC101856027
MyD88	IPR000157, IPR000488		5	LOC101855709, LOC101850562, LOC101855561, LOC101861518, LOC101858605
OrTIR	IPR000157		17	See supplemental file SFile3
SARM	IPR000157, IPR001660		3	LOC106011731, LOC101849069, LOC101850713
TOLLIP	IPR041799, IPR037301	IPR003892, IPR035892	1	LOC101845996
TAB1*	IPR001932		1	LOC101854409
TAB2/3*	IPR041911, IPR001876	IPR003892, IPR036443	1	LOC101854409

**Fig. 7:**
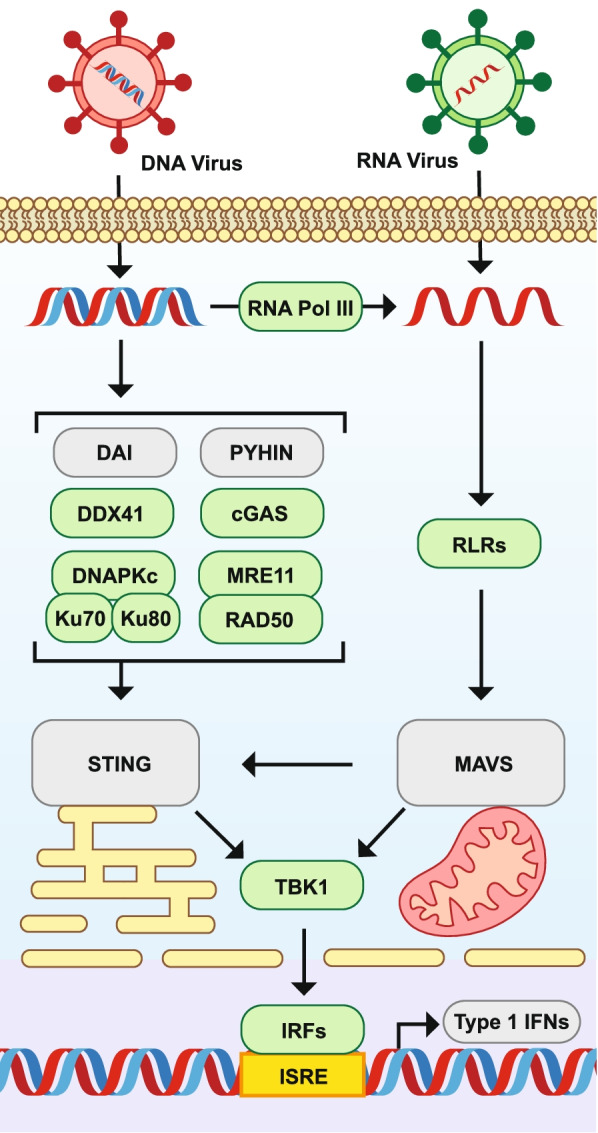
Conserved and absent elements of anti-viral signaling in *Aplysia californica*. In vertebrates, pattern recognition receptors for viral nucleotides signal through two major adapter proteins, Stimulator of Interferon Genes (STING) in the case of viral DNA and Mitochondrial Antiviral Signaling protein (MAVS) in the case of viral RNA. Both adapters facilitate the activation of Tank Binding Kinase 1 (TBK1) which activates Interferon Regulatory Factors (IRFs) that upregulate the transcription of Type 1 Interferons (IFN) to martial an antiviral response. *Aplysia californica* appears to lack orthologs of both STING and MAVS, as well as several viral DNA sensors found in vertebrates (grey filled boxes), suggesting possible divergent signaling pathways. Viral DNA sensors: DNA-dependent activator of IFN regulatory factors (DAI); Pyrin and hematopoietic interferon-inducible nuclear (HIN) domain (PYHIN) such as IFI16 and AIM2; DExD/H-box helicase 41 (DDX41); Cyclic GMP–AMP synthase (cGAS); DNA protein Kinase complex (DNAPK) comprised of DNAPK catalytic subunit (DNAKPKc), Lupus Ku autoantigen protein p70 (Ku70), and Lupus Ku autoantigen protein p80 (Ku80); and the complex made up of Meiotic recombination 11 (MRE11) and DNA repair protein RAD50. RNA sensors are RIG-I-like Receptors (RLR)

Notably, an ortholog of the downstream adaptor of RLRs, mitochondrial antiviral signaling protein (MAVS) could not be identified (Fig. 7). Similarly, an ortholog of Stimulator of Interferon Genes (STING), could not be identified (Fig. 7). STING acts an adaptor downstream of many DNA-damage associated PRRs including cGAS, DAI, PYHIN proteins, DNAPK, and the MIRE11-RAD50 complex [32].

#### E3 ubiquitin ligases

Ubiquitylation of various adaptor proteins and kinase complexes is a critical component of PRR signaling cascades downstream of many PRRs, notably TLRs, NLRs, and RLRs [47].

Tumor necrosis factor receptor (TNFR)–associated factor (TRAF) proteins play a key role in activating kinases during PRR signaling events [48]. In *Aplysia*, 6 TRAF-like proteins were identified: two resembling TRAF2 (LOC101856795 and LOC101844983), one resembling TRAF3 (LOC101846107), one resembling TRAF4 (LOC101859360, and two resembling TRAF6 (LOC101856862 and LOC101859878).

The LUBAC complex, comprised of proteins HOIP, HOIL1, and Sharpin, also plays a key role in RLR and TLR signaling, particularly in the activation of the IkB complex in NFkB signaling [49]. In *Aplysia*, putative orthologs of LUBAC complex components HOIP (LOC101849805) and HOIL1 (LOC101863809) were identified. Although an individual gene representing the third LUBAC component, Sharpin, was not identified, one of the 4 AcHOIL1 isoforms (XP_035825204.1) resembled Sharpin more then HOIL1 in conserved domain composition (i.e. lacking a TRIAD domain) and thus may represent a functional analog of Sharpin.

Another immune associated E3 ligase complex is comprised of SKIP1, cullin-1, and f-box proteins, or SCF [50]. In *Aplysia*, an ortholog of SKIP1 (LOC101854298), 6 putative cullins, and 60 f-box proteins, 14 of which contained WD40 domains (FBXW) and 29 of which contained leucine rich repeats (FBXL) were identified.

Other E3 ligases function to modulate or inhibit pro-inflammatory immune signaling. Pellino proteins modulates the activity of several immune associated kinases such as RIPKs and IRAKs [51]. In *Aplysia* only one Pellino gene (LOC101863733) was identified. Similarly, the diverse family of tripartite-motif contain proteins (TRIMs) regulate immune and proinflammatory signaling cascade via Ubiquitylation of various kinases [52]. TRIMs generally contain a b-box domain and a RING type zinc finger domain [53]. Twenty-two such genes in *Aplysia* were identified.

While the baculoviral Inhibitor of Apoptosis (IAP) repeat (BIR) family of proteins are known to modulate apoptosis, BIRC2/3, also known as cIAP1/2, are also known to modulate immune associated NFkB signaling [54]. The *Aplysia* genome encodes 22 BIR domain containing proteins, 11 of which also contained RING zinc finger domains that represent potential BIRC E3 ligases. Three of these also contained Death-like domains typical of cIAPs (LOC101851721, LOC101860034, and LOC101852760).

Signaling downstream of RLRs also depends on ubiquitylation via TRIMs and other E3 ligases such as MEX3C and RIPLET [55]. In *Aplysia*, a single potential MEX3C ortholog (LOC101854000) and two RIPLET-like genes (LOC101858054 and LOC101862807) were identified (Table 6).

**Table 6:** Putative E3 ubiquitin ligase genes identified in *Aplysia*. See Table 1 for table format description

	Required Domains		Genes	
Name	Strict	Relaxed	Count	Accessions
HOIL1	IPR044066, IPR000626, IPR027370, IPR001876	IPR026261, IPR001841, IPR036443	1	LOC101863809
HOIP	IPR002867, IPR018997, IPR032065, IPR041031, IPR044066, IPR001876	IPR026254, IPR001876	1	LOC101849805
IAPs	IPR001370		22	See supplemental file SFile3
MEX3C	IPR004088, IPR001841	IPR036612, IPR013083	1	LOC101854000
Pellino	IPR006800		1	LOC101863733
RNF134/RIPLET	IPR042723, IPR001841	IPR001870, IPR001841	2	LOC101862807, LOC101858054
SHARPIN	IPR031912, IPR001876, IPR029071	IPR026261, IPR000626, IPR036443	1	LOC101863809
SOCS	IPR035862, IPR001496	IPR036036, IPR000980	5	LOC101863556, LOC101857639, LOC101854668, LOC101856240, LOC101864191
TRAF	IPR001293, IPR002083, IPR001841	IPR043211, IPR002083	7	See supplemental file SFile3
TRIM	IPR000315, IPR001841		22	See supplemental file SFile3
TRIM25/65	IPR001870, IPR003877, IPR001841	IPR013320, IPR043136, IPR013083	2	LOC101862807, LOC101858054

#### Kinases

Interleukin-1 Receptor Associated Kinases (IRAKs) are serine/threonine kinases that complex with MyD88 via their Death-like domains and phosphorylate downstream adaptors in TIR signaling [56]. Two IRAK genes were identified, one (LOC101845636) is listed as IRAK4 on the NCBI, and the other (LOC101845840) assigned to the Pelle-family of IRAKs by PANTHER (PTHR24419:SF33).

In vertebrates, IRAK activation leads to activation of TRAF6, which ubiquitylates and activates the TAK1-TAB complex which contains the mitogen activate protein kinase (MAPK) kinase kinase 7 (MAP3K7/ TAK1) [57]. In *Aplysia* one ortholog of TAK1 which has 10 protein isoforms (LOC101852077) was identified. TAK1 phosphorylates two subsequent kinase chains: the IKK complex and mitogen activated protein kinases (MAPKs), that lead to translocation of transcription factors NFkB and AP-1 to the nucleus [45].

In canonical NFkB signaling, the IKK complex comprises IKKa/CHUK, IKKb, and IKKg/NEMO, and phosphorylates NFkB inhibitor proteins (NFkBIA, Cactus, etc.) to allow translocation of NFkB to the nucleus [58]. The non-canonical IKKs, IKKe and TBK1, complex together and subsequently with adaptors like SINTBAD or TANK to activate Interferon regulatory factors (IRFs) [59]. In *Aplysia* two canonical IKKS (LOC101856649, LOC106012158) and adaptor NEMO (LOC101858005) were identified, alongside two non-canonical IKKs (LOC101853683 and LOC101847449) that likely represent orthologs of TBK1 and IKKe. The only non-canonical IKK adaptor identified was a putative ortholog of NAP1 (LOC101852476) [60]. Orthologs for adapter proteins of another key immune associate kinase, TBK1, TBKBP1/SINTBAD and TANK, were not identified.

Parallel to IKKs, TAK1 and other MAP3Ks activate MAP2Ks (MKKs or MEKs), which activate MAPKs which in turn activate transcription factors, notably those in the AP-1 family [61]. In *Aplysia* several putative orthologs of several immune associated kinases in this three-tiered cascade were identified.

In addition to TAK1, one MAP3K in the Apoptosis signal-regulating kinase (ASK) family (LOC101862124) was identified, a MAP3K10 (LOC101864517), and a MAP3K12/13 (LOC101864115). Based on InterProScan annotation, it was not possible to identify orthologs of immune relevant MAP3Ks MAP3K14/NIK or MAP3K8/Tpl2. Annotation by PANTHER identified an additional 17 MAP3Ks.

MAP2Ks were difficult to identify purely using IPS domain annotation. Using the CDD analysis generated by IPS, a MAPK/ERK Kinase (MEK) with two isoforms (LOC101850764) was identified. Annotation by PANTHER further identified an additional 4 MAP2Ks (LOC101845548, LOC101846645, LOC101846695, LOC101860284).

Based on the MAPK-kinase conserved site (IPR003527), 6 MAPKs were identified, including orthologs of stress-activated kinases p38/MAPK14 (LOC100533304) and JNK, and an Extracellularly Regulated Kinase (ERK, LOC100533212). An additional p38 (LOC101857669) which lacks the conserved site but is identified as in the p38 family (IPR008352). PANTHER annotation identified 7 additional putative MAPKs that lacked the conserved site.

MAP kinase-activated protein kinase 2 (MK)-like gene (LOC101861689) and four MAPK phosphatases (MKP) that regulate MAPKs also were identified (Table 7).

**Table 7:** Putative immune associated kinase complex genes identified in *Aplysia*. See Table 1 for table format description. Note that IKKg/NEMO and NAP1 are adapters of canonical and non-cannonical IKK complexes respectively

	Required Domains		Genes	
Name	Strict	Relaxed	Count	Accessions
IRAK	IPR035533, IPR000719	IPR011029, IPR000719	2	LOC101845840, LOC101845636
IKKa/b	IPR041185, IPR000719		2	LOC101856649, LOC106012158
IKKg/NEMO	IPR034735, IPR032419	IPR032419, IPR034735	1	LOC101858005
non-canonical IKK (IKKe/TBK1)	IPR000719, IPR041309, IPR041087		2	LOC101853683, LOC101847449
NAP1	IPR019137		1	LOC101852476
MAPKs	IPR003527, IPR000719		13	LOC100533304, jnk, LOC101850222, LOC101859802, LOC101859802, LOC100533212
p38	IPR008352, IPR000719		2	LOC100533304, LOC101857669
MAP2Ks	IPR000719, IPR008271		5	LOC101850764, LOC101860284, LOC101846645, LOC101846695
ERK1/2	IPR008349, IPR000719		1	LOC100533212
JNK	IPR003527, IPR000719, IPR008351		1	jnk
MAP3Ks	IPR000719, IPR008271		20	See supplemental file SFile3
ASK	IPR043969, IPR025136, IPR000719		1	LOC101862124
TAK1	IPR017421, IPR001245, IPR000719		1	LOC101852077
MK2/ MAPKAPK2	IPR027442, IPR000719, IPR008271		1	LOC101861689
MKP	IPR008343		4	LOC101847406, LOC101846654, LOC101852280, LOC101847075
RIOK3	IPR000687, IPR017406		1	LOC101857430
JAK	IPR001245, IPR041381, IPR041155, IPR041046	IPR000719, IPR000299	1	LOC101861981

An ortholog of RIO Kinase 3 (RIOK3) which has been implicated as suppressor of RLR signaling but also as a critical adaptor for transcription factor IRF3 [62, 63] was also identified.

Notably, it was not possible to identify any orthologs of receptor-interacting serine/threonine protein kinases (RIPKs) which also play an important role in immune and inflammatory signaling [64].

#### Transcription factors

The ultimate action of PRR signal transduction cascades is the activation and translocation to the nucleus of pro-survival/pro-inflammatory transcription factors NFkB and AP-1 and interferon (IFN) regulatory factors (IRFs) [65].

Nuclear factor kappa beta (NFkB) transcription factors are the quintessential and evolutionarily conserved immune/inflammation transcription factors [66]. Based on Rel homology domains (IPR032397 and IPR011539) a p105-like protein with two isoforms (LOC101855313) and a RelB/Dorsal-like (LOC101860399) protein were identified. A putative ortholog of Nuclear factor of activated T cells (NFAT, LOC101858233), which are a related class of immune transcription factors, was also identified. NFkB proteins are characteristically bound by inhibitor proteins (NFKBI/IkB) which are the target of IKKs. *Aplysia* contains putative orthologs of NFKBIA with 4 isoforms (LOC101852802) and of NFKBI Cactus (LOC101863680).

Just as NFkB is the target of the IKK branch of immune signaling, activator protein 1 (AP-1) is the downstream target of the MAPK cascade. AP-1 is a dimeric transcription factor made up of any two of a related group of proteins families known as JUN, FOS, MAF, and ATF [67]. In Aplysia, 8 AP-1 type transcription factors were identified, including one identified as ATF2-like (LOC101856191). Homology search further identified the *Aplysia* c-Jun gene (LOC101863323).

Parallel to NFkB and AP-1, IRFs are activated by immune signaling such as TLRs or cytosolic nucleic acid sensors and facilitate the translation of interferon cytokines and interferon stimulated genes (ISG) to counteract infection [68]. Seven IRFs were identified: 2 IRF1/2-like that lack SMAD domains (LOC101855936 and LOC101850966) and 5 SMAD domain containing IRFs. Translocation of these transcription factors is facilitated by major vault proteins (MVP) [69], 4 of which were identified in *Aplysia* (Table 8).

**Table 8:** Putative transcription factor genes identified in *Aplysia*. See Table 1 for table format description. Note that MVP and IkB are modulators of transcription factor activity

	Required Domains		Genes	
Name	Strict	Relaxed	Count	Accessions
β-Catenin	IPR013284		1	LOC100533383
AP-1	IPR000837, IPR004827		8	See supplemental file SFile3
JUN	IPR002112, IPR004827		1	c-Jun
IRF	IPR001346		7	See supplemental file SFile3
NFkB	IPR033926, IPR011539	IPR032397, IPR011539	3	LOC101855313, LOC101858233, LOC101860399
IkB	IPR002110, IPR038753	IPR038753, IPR036770	1	LOC101852802
MVP	IPR041139, IPR021870	IPR043023, IPR036013	4	LOC101847145, LOC101848074, LOC101861920, LOC101860982
STAT	IPR000980, IPR013800, IPR013801, IPR013799	IPR013801, IPR001217	1	LOC101861517

Successful activation of NFkB, AP-1, and IRFs transcription factors leads to the production of cytokines that recruit another family of critical immune transcription factor: Signal Transducer and Activator of Transcription (STAT) proteins. Cytokine receptors for Interleukins and Interferons complex with Janus Kinases (JAKs) which phosphorylate STAT transcription factors to promote transcription of interferon stimulated genes (ISGs) [70]. This pathway is then autoinhibited by Suppressor of Cytokine Signaling (SOCS) proteins. In Aplysia, a single JAK (LOC101861981), a single STAT (LOC101861517), and 5 SOCS (LOC101845832) were identified.

Another major transcription factor that plays a role in immune response is the pro-proliferative Myelocytomatosis oncogene (MYC). While MYC is generally known for its role in modulation of the cell cycle and cellular proliferation, it has also been demonstrated to play a role in not only immune cell proliferation but also immunomodulation [71]. Conserved domain search failed to identify an *Aplysia* MYC based on conserved leucine zipper (IPR003327) and n-terminal domains (IPR002418). Homology search also failed to identify an *Aplysia* ortholog of MYC. However, 52 genes were identified with a myc-type basic helix-loop-helix (bHLH) domain typical of transcription factors related to MYC, leaving the possibility a MYC-like transcription factor may yet be identified.

#### Cytokines

Successful activation of immune receptor transduction results in the secretion of pro-inflammatory cytokines [72]. Activation of pro-inflammatory cytokine receptors and associated pathways then stimulates the proliferation of immune cells in an inflammatory immune response [73]. Seven putative orthologs of the pro-inflammatory cytokine Macrophage migration inhibitory factor (MIF), a progranulin ortholog (LOC101846478), 4 putative tumor necrosis factors (TNF), and 4 interleukin-17 -like cytokines (IL-17) was identified. Three putative IL-17 receptors and 15 TNF receptor-like genes also were identified (Table 9).

**Table 9:** Putative cytokine signaling associated genes identified in *Aplysia.* See Table 1 for table format description

	Required Domains		Genes	
Name	Strict	Relaxed	count	Accessions
ADAM	IPR001590		25	See supplemental file SFile3
CRADD	IPR042148, IPR037926	IPR001315, IPR000488	1	LOC101855728
FADD	IPR001875, IPR000488	IPR016729	1	LOC101861195
Granulins	IPR000118	IPR037277	1	LOC101846478
IL-17	IPR010345, IPR029034		4	LOC101857089, LOC101857312, LOC101846730, LOC101857050
IL17R	IPR013568, IPR039465	IPR013568	3	LOC101850817, LOC101849802, LOC101852736
LITAF	IPR006629		6	See supplemental file SFile3
MIF	IPR019829	IPR001398	7	See supplemental file SFile3
TNF	IPR006052		4	LOC101857902, LOC101854405, LOC101845807, LOC101861555
TNFR	IPR001368		15	See supplemental file SFile3

TNF receptors associate with adaptor proteins via Death domains (DD) to transduce signaling downstream to the pro-inflammatory NFkB signaling pathway or to the pro-apoptotic caspase pathway. These DD containing signaling adaptors are TRADD, CRADD/RAIDD, and FADD. Putative CRADD (LOC101855728) and FADD (LOC101861195) orthologs were identified, but not TRADD.

Although not adapter proteins per se, A disintegrin and metalloproteinase (ADAM) proteins have been implicated in immune function, notably in cytokine signaling, particularly of TNF and Interleukins [74]. Twenty-five ADAMs were identified based on conserved peptidase domain (IPR001590).

#### Apoptosis

Apoptosis is the programmed self-destruction of cells and plays key roles in diverse biological processes such as development and the immune response. In mollusks, apoptosis is believed to play a critical role in the immune response by destroying pathogen infected cells and limiting the spread of infections without inducing inflammation [75]. Indeed, apoptosis serves as a key mechanism for slowing the spread of viral infections across taxa [76]. In addition to BIR containing apoptosis inhibitors mentioned in previous sections, several genes in the Bcl-2 family of proteins also act as important modulators of apoptosis [77]. In *Aplysia*,6 Bcl-2 family proteins, including putative ortholog of anti-apoptotic Bcl-2 (LOC101848156), as well as pro-apoptotic proteins Bax (LOC101854270), Bak (LOC101851280), and Bok (LOC101854628) were identified. Two potential Fas Apoptosis Inhibitor Molecules (FAIM, LOC101863121 and LOC106012994), which act as apoptosis inhibitors and play a role in IFN signaling [78] were identified. Apoptosis signaling is most notably mediated through pro-inflammatory and pro-apoptotic proteases called caspases [79]. Eight caspase genes were identified. Other apoptotic regulators Death factor and mitochondrial metabolism regulator AIF (LOC100533557) [80] and IAP inhibitor and pro-apoptotic factor HTRA2 [81] was also identified (Table 10).

**Table 10:** Putative apoptosis associated genes identified in *Aplysia.* See Table 1 for table format description

	Required Domains		Genes	
Name	Strict	Relaxed	count	Accessions
AIF	IPR029324, IPR023753	IPR029324, IPR036188	1	LOC100533557
Bcl2-like	IPR026304, IPR003093	IPR002475	6	See supplemental file SFile3
Caspase	IPR015917		8	See supplemental file SFile3
FAIM	IPR038513	IPR010695	2	LOC101863121, LOC106012994
HTRA2	IPR041489, IPR001478, IPR001940		1	LOC101848371

### Effectors

#### Anti-microbial

In mollusks, often the first line of defense against pathogens is the mucus, which contains a diverse array of antimicrobial compounds including lectins and mucins [75, 82]. In addition to previously discussed lectins, the *Aplysia* genome also encodes 13 mucins. Among the most ancient antimicrobial proteins are those that punch holes in the cellular membranes of pathogens, called pore forming proteins (PFP) [83, 84]. In *Aplysia*, 14 Aerolysin type, 5 ETX/MTX-2 type, and three MACPF containing MPEG-like pore forming proteins were identified. Lysozymes are similarly ubiquitous components of the innate immune system that act to lyse pathogen membranes [85]. In *Aplysia* three lysozymes were identified (Table 11). Other antimicrobial proteins known from bivalves such as defensins, mytilins, or mytimicins were not identified.

**Table 11:** Putative anti-microbial genes identified in *Aplysia.* See Table 1 for table format description

	Required Domains		Genes	
Name	Strict	Relaxed	count	Accessions
Aerolysin	IPR005830		14	See supplemental file SFile3
ETX/ MTX-2	IPR004991		5	LOC101857663, LOC101857969, LOC101846908, LOC101858001, LOC106011851
MACPF	IPR020864		3	LOC101845147, LOC101864334, LOC101861925
Mucin	IPR000082	IPR036364	13	See supplemental file SFile3
Lysozyme	IPR019799	IPR023346	3	LOC101864662, LOC106013490, LOC106014021

#### Anti-viral

In the case of virus infection specifically, cells mobilize a diversity of antiviral proteins that hamper virus entry, uncoating, replication, translation, assembly, and release [29]. Two dynamin family Mx-like proteins (LOC101848065 and LOC101863954), one RNA Specific Adenosine Deaminase (ADAR) with three isoforms (LOC101855902), four viperins/RSAD, 8 caveolins, 5 Gilt-like proteins, three APOBEC3-like proteins, and 10 Interferon-induced transmembrane protein/Dispanin family (IPR007593) proteins were identified. OrthoFinder homology search identified one further potential ADAR gene (LOC101863647), which contains dsRNA binding domains (IPR014720) and adenosine deaminase/editase domains (IPR002466) but lacks a Z-binding domain (IPR042371). Orhtologs to other antiviral proteins including tetherins, SAMHD, Zinc finger antiviral protein (ZAP), IFI41, or RNAse-L orthologs were not identified.

Similarly to DNA damage response genes being implicated as PRRS, poly(ADP-ribose) polymerases (PARPs) which are associated with DNA damage response have also been demonstrated to have broad spectrum anti-viral and pro-inflammatory effects [86, 87]. In *Aplysia*, 17 PARP genes were identified (Table 12).

**Table 12:** Putative anti-viral genes identified in *Aplysia.* See Table 1 for table format description

	Required Domains		Genes	
Name	Strict	Relaxed	count	Accessions
ADAR	IPR002466, IPR044456, IPR044457, IPR014720, IPR042371	IPR002466, IPR014720, IPR042371	1	LOC101855902
APOBEC3	IPR002125, IPR016192	IPR040551	3	LOC101861380, LOC101858634, LOC101854808
CAV	IPR018361	IPR001612	8	See supplemental file SFile3
Gilt	IPR004911		5	LOC101846476, LOC101863576, LOC101845647, LOC101848961, LOC101846527
IFITML	IPR007593		10	See supplemental file SFile3
Mx	IPR003130, IPR001401, IPR000375, IPR045063	IPR020850, IPR030381, IPR027417	2	LOC101848065, LOC101863954
PARPs	IPR012317		17	See supplemental file SFile3
RSADs	IPR007197, IPR013785		4	LOC101856483, LOC101856246, LOC101847430, LOC101847031
ZNFX1	IPR041677, IPR041679, IPR000967, IPR016024	IPR045055, IPR041677, IPR041679	3	LOC101857249, LOC101862822, LOC101851452

#### RNAi

RNA interference (RNAi) is another potent antiviral mechanism of particular importance in arthropods [88]. Key components of the RISC RNAi complex, including Dicer (LOC101848232), two argonaute proteins (LOC101846762 and LOC101863465), and two additional PIWI domain containing proteins that lack argonaute linker domains were identified (Table 13). An ortholog of RISC-loading complex subunit TARBP2 could not be identified via InterPro conserved domains alone. OrthoFinder homolog search, however, identified LOC101846211, named TARBP2 on the NCBI, as a potential TARBP2. LOC101846211 contains several dsRNA binding domains (IPR014720) typical of TARBP2, as well as a Staufen c-terminal (IPR032478).

**Table 13:** Putative RNAi genes identified in *Aplysia.* See Table 1 for table format description

	Required Domains		Genes	
Name	Strict	Relaxed	count	Accessions
Argonaute	IPR032473, IPR032474, IPR003100, IPR003165, IPR014811, IPR032472	IPR003165, IPR036085, IPR032473	2	LOC101863465, LOC101846762
Dicer	IPR044441, IPR000999, IPR003100	IPR014720, IPR038248, IPR036085, IPR036389	1	LOC101848232
PIWI	IPR003165, IPR014811, IPR003100	IPR003165	4	LOC101852400, LOC101863465, LOC101862357, LOC101846762
TRBP*	IPR044469, IPR044470, IPR044471	IPR014720, IPR028605	1	LOC101846211

#### ROS and Antioxidants

A prime function of immune cells is the capacity to recognize, phagocytose, and destroy pathogens during an immune response. Phagocytosed pathogens are destroyed by the generation of reactive oxygen species (ROS). ROS also play a role in potentiating pro-inflammatory signaling cascades during immune signaling [89]. In *Aplysia* a diverse set of ROS generating enzymes were identified, including: 3 dual oxidases (DUOX) and 2 associated assembly factors (DUOXA), 7 nitric oxide synthases (NOS), 5 NADPH oxidases, and one peroxidasin (LOC100533347). Furthermore, 20 L-amino-acid oxidases (LAAO) were identified, among which was antimicrobial peptide Aplysianin-A (LOC100533295) [90].

Due to the cytotoxic nature of ROS, levels of reactive species are tightly regulated in the cell via antioxidant enzymes. Additionally, antioxidant enzymes have also been demonstrated to act as immunomodulators of proinflammatory cytokines [91]. In *Aplysia,* a robust complement of antioxidant enzymes was identified, including: 8 Cu-Zn superoxide dismutases (SOD), one Fe-Mn superoxide dismuates (MnSod, LOC101852344), two catalases (LOC101854685 and LOC101860167), and 14 peroxiredoxins. In the glutathione antioxidant pathway, 3 glutathione peroxidases, 8 glutaredoxins, a glutathione reductase (LOC101847425), and 68 glutathione S-transferases were identified (Table 14).

**Table 14:** Putative ROS generating and antioxidant genes identified in *Aplysia.* See Table 1 for table format description

	Required Domains		Genes	
Name	Strict	Relaxed	count	Accessions
DUOX	IPR011992, IPR002048, IPR013112, IPR017927, IPR013130, IPR039261, IPR019791, IPR017938		3	LOC101859126, LOC101855136, LOC101857144
DUOXA	IPR018469		2	LOC101855368, LOC106013841
LAAO	IPR002937		20	See supplemental file SFile3
NOS	IPR004030, IPR008254	IPR008254	7	See supplemental file SFile3
NOX	IPR017927, IPR013130, IPR013121	IPR000778	5	LOC101851552, LOC101854582, LOC101859126, LOC101855136, LOC101857144
PRXDN	IPR034824	IPR029610	1	LOC100533347
CAT	IPR011614		2	LOC101854685, LOC101860167
GPX	IPR029760	IPR000889	3	LOC101845824, LOC101850882, LOC106012346
GRX	IPR002109		8	See supplemental file SFile3
GSR	IPR023753, IPR004099, IPR006322	IPR006322, IPR036188, IPR016156	1	LOC101847425
GST	IPR004046, IPR004045	IPR010987	68	See supplemental file SFile3
MnSOD	IPR019832, IPR019831		1	LOC101852344
PRDX	IPR000866	IPR013766, IPR019479	4	LOC100533273, LOC101852808, LOC101852939, LOC101861906
PRX	IPR019791	IPR037120	14	See supplemental file SFile3
SOD	IPR001424		8	See supplemental file SFile3
TRXDC	IPR013766		38	See supplemental file SFile3

#### Heat shock proteins

Stress signaling during an immune response is known to induce the transcription of heat shock protein (HSP), which also have been suggested to have immunomodulatory function [92]. In *Aplysia,* many HSPs across several families were identified (Table 15), including: 20 HSP20s, 12 HSP70s, 4 HSP90s, and one heat shock factor (HSF, LOC101851429).

**Table 15:** Putative heat shock proteins genes identified in *Aplysia.* See Table 1 for table format description

	Required Domains		Genes	
Name	Strict	Relaxed	count	Accessions
HSF	IPR003594, IPR000232	IPR027725	1	LOC101851429
HSP20	IPR002068	IPR008978	25	See supplemental file SFile3
HSP70	IPR018181	IPR013126	12	See supplemental file SFile3
HSP90	IPR019805, IPR020575, IPR003594	IPR001404	4	LOC101845707, LOC101859343, LOC101861407, LOC101859248

#### Melanization

In arthropods, part of the immune response involves the production of toxic quinone compounds, wound healing factors, and pathogen encapsulating melanin in a process known as melanization. Although most well described in arthropods, melanization as an immune response has also been described in several bivalves [93] and in the gastropod *B. glabrata* [94]. While 5 laccases and 6 tyrosinases that participate in melanization were identified in *Aplysia*, an ortholog of the critical Phenol Oxidase central to arthropod melanization could not be identified.

#### Complement system

A major component of the vertebrate immune response is the complement system, which facilitates the formation of the pathogen destroying membrane attack complex (MAC) and the recruitment of phagocytic cells [95]. The complement system is triggered after PAMP/DAMP binding to one of three lectins: Complement 1 (C1q), MBL, or fibrinogen and collagen domain containing lectins (ficolins) [96]. While neither MBL nor ficolins were identified in *Aplysia*, 11 C1q domain containing genes (C1qDC) were identified. In addition, two orthologs for C1q receptor Multiple epidermal growth factor-like domains protein 10 (MEGF10, LOC101848617 and LOC106013429) were identified. Activation of complement PRRs recruits a group of thioester containing proteins called complement factors.

Thioester containing proteins (TEPs) play an important role in pathogen defense across the animal kingdom. TEPs are subdivided into complement factors in the complement system and alpha 2 Macroglobulins (a2Ms) which act as protease inhibitors [96]. In *Aplysia* 13 TEPs based on conserved thioester domains (IPR011626) were identified. Two of these, LOC101848728 and LOC101862906, could be classified as complement factors C3 and C4-A respectively based on conserved domains. LOC101854858 and LOC101861335 were further classified as CD109-like proteins based on conserved domains. On the other hand, a factor B ortholog (Bf) based on conserved domains could not be identified. However, unlike vertebrate Bf which contains type A von Willebrand factor, Sushi, and Trypsin domains, the *C. gigas* gene identified as Bf by Zhang et al. 2015 contains von Willebrand type A (IPR002035), Sushi (IPR000436), EGF (IPR000742), and Pentraxin domains (IPR001759) [3]. Based on these domains, an *Aplysia* Bf (LOC101854327) was identified from among the *Aplysia* genes identified as Pentraxins (Table 16).

**Table 16:** Putative complement system component genes identified in *Aplysia.* See Table 1 for table format description

	Required Domains		Genes	
Name	Strict	Relaxed	count	Accessions
A2M	IPR001599		13	See supplemental file SFile3
C1qDC	IPR001073		11	See supplemental file SFile3
C3/4/5	IPR001134, IPR009048, IPR011626	IPR008993, IPR036595	2	LOC101848728, LOC101862906
CD109	IPR009048, IPR011625, IPR041813, IPR013783, IPR001599, IPR002890, IPR041555	IPR036595, IPR011626, IPR011625, IPR041813, IPR013783, IPR001599	2	LOC101854858, LOC101861335
Bf	IPR002035, IPR000436, IPR001254 (IPR000436, IPR000742, IPR002035, IPR001759)	IPR011360	1	LOC101854327
MEGF10	IPR001881, IPR000742, IPR011489		2	LOC101848617, LOC106013429
TEP	IPR011626		13	See supplemental file SFile3

## Discussion

In this in silico analysis, a robust immune complement in the *Aplysia* genome was proposed. This includes PRRs and adaptor proteins, signal transducers, transcription factors, and subsequent cytokine signaling components [97–99]. Among these are NFkB transcription factors, the IKK complex, a diverse compliment of MAPKs and associated kinases, the TAB-TAK1 complex, E3 Ubiquitin ligases like Pellino, TRAFs, and the LUBAC complex, and a diverse collection of TIR domain adaptors and receptors.

**Fig. 8:**
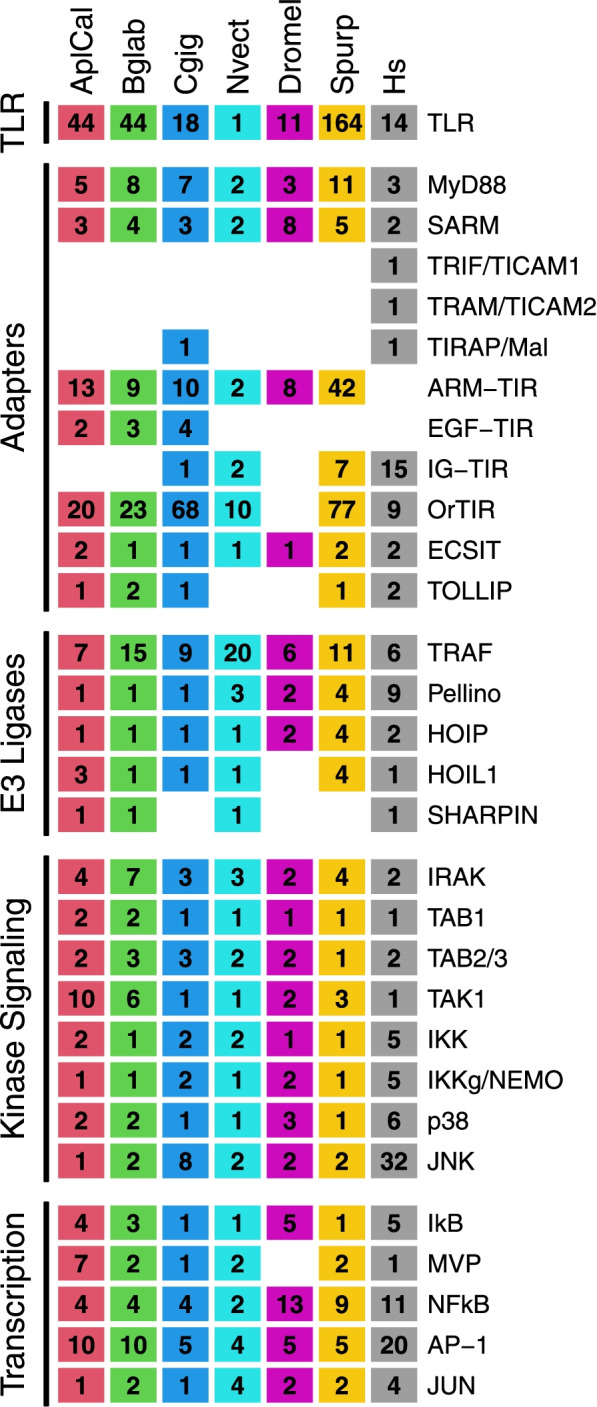
Orthologous components of the TLR signaling cascade in *Aplysia californica* and other animals. Immune genes of interest were extracted from a custom InterProScan annotation of the *Aplysia californcia* RefSeq predicted protein models (AplCal, GCF_000002075.1) and publicly available InterPro annotations of proteins from the UniprotKB data base for *Biomphalaria glabrata* (Bglab, UP000076420_IPS), *Crassosteraea gigas* (Cgig, UP000005408), *Nematostella vectensis* (Nvect, UP000001593), *Drosophila melanogaster* (Dromel, UP000000803), *Strongylocentrotus purpuratus* (Spurp, UP000007110)*,* and human (Hs, UP000005640) based on InterPro domains detailed in supplementary data Supplemental File SFile5. Results were further refined using sequence similarity search among listed proteomes and predicted protein models using OrthoFinder and BLASTP. Columns of the table represent a single species, while rows represent proteins known to play key roles in TLR signaling in mammals. Proteins are grouped according to their functional roles in TLR signaling: TLR receptors, Adapter proteins like MyD88, E3 Ubiquitin ligases, Kinase signaling complexes and components, and Transcription factors and associated proteins. Numbers in each cell represent the number of protein hits to each protein type, and thus differ from gene level numbers present in the main text. Note that hits are to Uniprot KB proteomes and as such differ from previously reported numbers for *C. gigas* and *S. purpuratus* which used predicted gene models. *Aplysia* protein numbers for major components are broadly similar to other animals, although TLRs appear to be highly diversified compared to mammals and arthropods, but still fewer in number than in *S. purpuratus* and previously reported numbers for *C. gigas* (83 genes vs the 18 identified proteins with out methods in Uniprot KB). Similarly, TLR/Interleukin-1 receptor domain (TIR) containing adapters are much more diverse in *Aplysia* compared to human, but again less diverse than that of *S. purpuratus* and *C. gigas*. Toll-like receptor (TLR); Myeloid differentiation primary response protein (MyD88); NAD(+) hydrolase SARM1 (SARM); TIR domain-containing adapter molecule 1 (TRIF/TICAM1); TIR domain-containing adapter molecule 2 (TRAM/TICAM2); Toll/interleukin-1 receptor domain-containing adapter protein (TIRAP/Mal); Armadillo fold and TIR domain containing proteins (ARM-TIR); Epidermal growth factor and TIR domain containing proteins (EGF-TIR); Imunoglobulin and TIR domain containing proteins (IG-TIR); Orphan TIR proteins (OrTIR); Evolutionarily conserved signaling intermediate in Toll pathway (ECSIT); Toll-interacting protein (TOLLIP); TNF receptor-associated factors (TRAF); E3 ubiquitin ligase Pellino (Pellino); E3 ubiquitin-protein ligase RNF31 (HOIP); RanBP-type and C3HC4-type zinc finger-containing protein 1 RBCK1 (HOIL1); Sharpin (SHARPIN); Interleukin-1 receptor-associated kinase 1 (IRAK); TGF-beta-activated kinase 1 and MAP3K7-binding protein 1 (TAB1); TGF-beta-activated kinase 1 and MAP3K7-binding protein 2/3 (TAB2/3); MAP3K7/TGF-beta-activated kinase 1 (TAK1); Inhibitor of nuclear factor kappa-B kinase subunit alpha (IKK); NF-kappa-B essential modulator (IKKg/NEMO); p38 MAPK/ MAPK14 (p38); Mitogen-activated protein kinase 8 (JNK); Inhibitor of NFkB (IkB); Major Vault Protein (MVP); Nuclear factor kB (NFkB); Activating protein 1 family (AP-1); Activating protein 1 JUN (JUN)

**Fig. 9:**
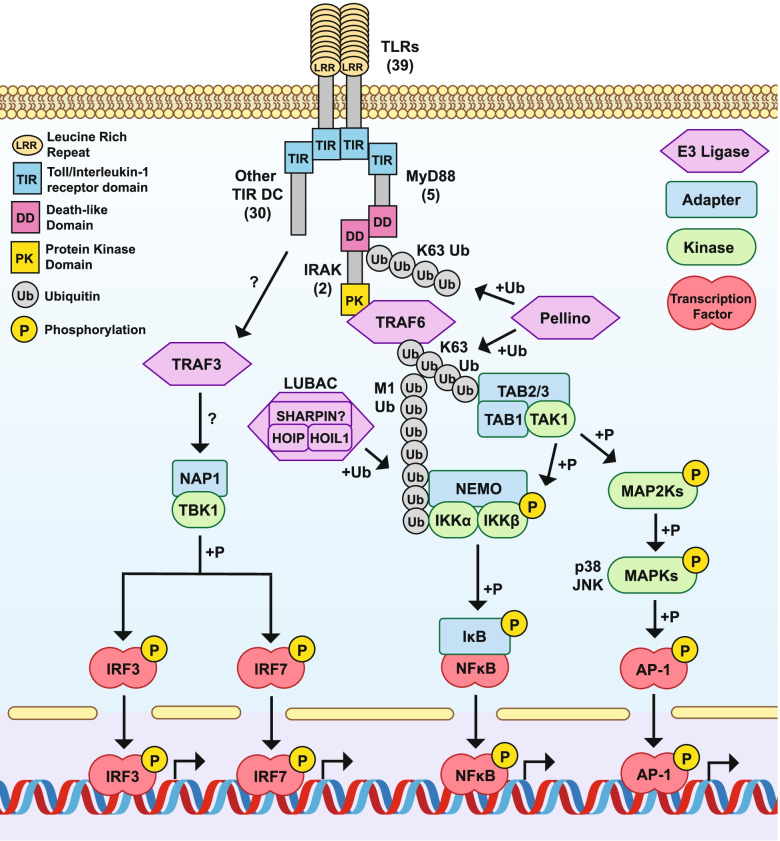
Potentially Toll-like receptor signaling cascade in *Aplysia californica*. Based upon conservation of key components of the vertebrates TOLL-like receptor (TLR) cascade and conserved function of those components in other mollusks, we propose a vertebrate-like TLR signaling cascade in *Aplysia californica*. The Aplysia genome encodes putative orthologs of all major components of the classical TLR singaling cascade, including a diversity of TLRs, adapter protein MyD88, two MyD88 interacting IRAKs, E3 ligases and scaffolding protein, E3 ligase Pellino, E3 ligase LUBAC complex comprised of HOIP, HOIL1, and SHARPIN, the Tak1 kinase complex comprising of TAB1, TAB2/3, and TAK1, the IkB Kinase (IKK) complex made up of IKKa, IKKb, and IKKg/NEMO, several MAP kinases, and transcription factors in the NFkB, interferon regulatory factor (IRF), and AP-1 family. The Aplysia genome also encodes for elements of non-canonical TLR/NFkB singaling including TRAF3, non-canoninical IKK TABK1 and associated adapter NAP1. A broad suit of non-Myd88 TIR domain containing proteins suggests a diverse suit of alternative TLR signaling cascades that have yet to be described

Functional studies in *C. gigas* have demonstrated that the oyster TLRs function as immune sensors and signal through MyD88 down to NfKB as in vertebrates [100]. Similarly. Studies have shown that the oyster IKK complex does indeed form from two IKKs and adapter NEMO as in mammals. Furthermore, oyster IKK interacts with TRAF6 and MyD88 and induces the transcription of NF-kB and IRF target genes, likely through NFkB and IRF8 orthologs [101]. Indeed, mollusks in general appear to retain a TLR signaling cascade like that of vertebrates [98]. As in other mollusks, the *Aplysia* genome encodes essentially all the major components of the canonical TLR signaling cascade (Figs. 8). Given the broad conservation of genes from the TLR cascade in mollusks, and the conserved function of many of those genes as described in oyster, *Aplysia* possibly also retain a vertebrate-like TLR signaling cascade (Fig. 9). However, molluscan TLRs appear to have undergone massive expansion compared to vertebrates, suggesting a more diverse TLR signaling pool beyond the canonical pathway conserved with vertebrates.

Previously, a survey of the *C. gigas* genome demonstrated a massive expansion of the short protostome type TLRs and a smaller expansion of TLRs containing leucine-rich repeat c-terminals. This expansion led to a comparatively high TLR count, though was different in type compared to *S. purpuratus* which exhibited an expansion but in vertebrate-type TLRs. These TLRs were upregulated in response to boiotic challenge, suggesting a unique molluscan immune TLR cascade. Compared to *C. gigas* and *S. purpuratus*, *Aplysia* is comparatively poor in TLRs (39 in *Aplysia* vs 83 in *C. gigas* and 222 in *S. purpuratus*). Nevertheless, like other invertebrates, the *Aplysia* genome encodes many more TLRs than in vertebrates. While direct comparison to previously reported *C. gigas* numbers was not possible due to differences in custom vs reference protein number for *C. gigas*, the neighbor joining tree generated in this study corroborates earlier results that demonstrate that the massice expansion of TLRs in *S. purpuratus* and in mollusks represent distinct expansion of different TLR families [3]. Interestingly, unlike the *C. gigas* TLRs, the gastropod TLRs predominantly clustered within the proteosome specific branch of the tree, with the majority clustering in the mollusk specific branch. These results agree with OrthoFinder results which suggest most of the gastropod TLRs form a unique group apart from arthropods, veterbrates, and even bivalves. This may suggest a unique gastropod TLR expansion that may hint at unique gastropod TLR signaling pathways.

Concomitantly, The *Aplysia* genome further contains a diversity of TIRDC that may represent novel or expanded TLR signaling adaptors. Among these are several MyD88 and SARM-like TIR adaptor proteins, both of which are known from diverse lineages and are considered the most ancient of the TLR adapters [44, 102]. However, many contain domains unknown from vertebrate TIRDC adapter. The presence of Armadillo-like folds in several *Aplysia* TLRs and TIRDCs may represent a novel TLR signaling cascade. Similarly, the presence of ROCO domains in two AcTLRs, two AcMyD88s, and an AcTIRDC may represent another novel GTPAse TLR signaling cascade. The inability to identify orthologs of TIR adapters TICAM1/TRIF and TICAM2/TRAM was expected as this MyD88-independent TLR signaling cascade arose only in the chordate lineage [102]. This suggests that the TRIF/TRAM/TBK1 signaling axis of some vertebrate TLRs is absent in *Aplysia*, or that another set of TIRDC adaptors fill an analogous role of TICAMs in non-canonical NFkB signaling. Downstream of TIRDCs, *Aplysia* retains two IRAKs as in other invertebrates, like the arthropod *Drosophila* and basal chordate *Amphioxus*. Given that these two IRAK proteins transduce TIR activation in different ways between taxa, it is unclear how *Aplysia* IRAKs interact with *Aplysia* TRAFs or Cactus orthologs [56].

Unlike in arthropods, *Aplysia* also appears to retain several key components of the complement system, including C1q lectins, many thioester proteins including factors C3 and C4, and factor B. While the complement system has substantially changed in the vertebrate lineage since the emergence of jawless fish, evidence suggests that the basic components of this pathway have ancient roots in early metazoa [103]. In this context, the *Aplysia* complement system likely serves to opsonize pathogens and induce inflammation [104]. In oyster, the C1q domain containing family underwent an extensive expansion like the TLRs [3]. In comparison, Aplysia is comparatively poor in C1qDC proteins, both compared to oyster and human.

Of particular interest for future studies of the *Aplysia Abyssovirus*, the *Aplysia* genome encodes many anti-viral genes. Among these are 5 Gamma-interferon-inducible lysosomal thiol reductases (Gilt/IFI30), which are key ISGs that inhibit viral entry in mammals [105]. Crustacean orthologs of Gilt have been demonstrated to have conserved viral entry-inhibiting function [106]. Although reported as an Mx ortholog, DQ821497 of *Haliotis discus* appears to be an ortholog of *Aplysia* Gilt based on homology and conserved domains. This abalone Gilt has been demonstrated to be induced by viral RNA mimic Poly I:C, suggesting conserved antiviral function of gastropod Gilt orthologs as well [107]. Vertebrate Mx proteins are unrelated to Gilt proteins and are instead related to dynamins and serve to prevent entry and replication of a diverse array of viruses [108]. Several dynamin-like genes were identified in *Aplysia*, but tree-based result from OrthoFinder suggests these *Aplysia* dynamins are more like vertebrate dynamins than vertebrate Mx proteins, and so may not exhibit any antiviral activity.

The *Aplysia* genome also encodes orthologs of antiviral deaminases ADAR and APOBEC3. These ISGs inhibit viral replication and function by introducing point mutations into viral genomes [109, 110]. RNA editing of viral genomes by molluscan ADAR genes has been demonstrated to play an important role in the immune response to malacoherpseviruses [111]. The *Aplysia* genome also encodes a large complement of PARP proteins, which have been demonstrated to have strong anti-viral action against a diverse suite of viruses in mammals [112]. If these defenses are insufficient to protect cells from active infection, the *Aplysia* genome also encodes several viperins to prevent viral spread. Viperins are ISGs that serve to inhibit viral budding and release from lipid bodies in diverse taxa [113]. Conservation of function for molluscan viperins has been demonstrated in oysters [114]. Although the *Aplysia* genome encodes an immune compliment with substantial conservation with known innate immune genes, several major immune pathways or components appear to be missing.

Despite containing two non-canonical IKKs, the *Aplysia* genome lacks many of the components associated with TBK1 signaling in vertebrates. The adaptor proteins STING and MAVS, neither of which have identifiable orthologs in *Aplysia*, are both known to act as scaffolds that facilitate TBK1 activation of IRF3 in response to antiviral PRR activation [115].

While present in bivalves, the absence of STING has been described previously in heterobranch gastropods like *Aplysia* [98]. STING acts as an adaptor that facilitates phosphorylation of IRF3 by TBK1 in a diverse suit of nucleotide sensing pathways, many of which are either absent in *Aplysia* (DAI, PYHIN proteins), or non-productive in the heterobranch lineage (cGAS). The *Aplysia* genome does however encode for the MRN and DNAPK complexes, which have also been described as able to activate TBK1 in a STING dependent and independent fashions. *Aplysia* also lack the upstream components of TLR associated TBK1 signaling; notably TICAM proteins and TBK1 adaptors TANK and SINTBAD that form an analogous signaling cascade to the MyD88-TAB-TAK1 axis. TRADD, which is also known to complex with TRAF2/3 and activate the TANK-TBK1-IRF axis is also absent. Interestingly, *Aplysia* does retain an ortholog of RIOK3, which has been described as an essential adaptor of TBK1 and as an inhibiter of RLR signaling [62, 63, 116]. Given the diversity of yet undescribed TIRDC proteins in *Aplysia*, perhaps a convergent non-canonical IKK signaling pathway has emerged in the heterobranch lineage.

Given the absence of STING in heterobranchs, despite its presence in the bivalve lineage, it is perhaps not surprising that *Aplysia* also lack a MAVS ortholog [117, 118]. Nevertheless, the *Aplysia* genome does still include 3 putative RLRs that act upstream of MAVS in vertebrates and presumably bivalves. Notably, the *Aplysia* RLRs lack any CARD or DLD which act to form complexes between RLRs and MAVS in canonical RLR signaling [119]. This condition of *Aplysia* RLRs is conserved with those in *Biomphalaria*, suggesting a functional divergence of heterobranch RLRs from those in bivalves. Perhaps heterobranch RLRs follow a similar pattern to the highly divergent immunoglobulin domains of VIgLs, where the functional domain is conserved but so highly divergent that it is difficult for automated systems to detect. Perhaps a similarly highly divergent MAVS-like protein has yet to be identified in heterobranchs, or a novel signaling axis for RLRs has emerged in the absence of MAVS. Future studies coprecipitating *Aplysia* RLRs with interacting proteins may be able to uncover such a highly divergent MAVS, if it indeed exists.

Another notably absent immune axis is that of OAS1 and its downstream effector RNAse L. OAS acts as a sensor for a diverse repertoire of viruses, including RNA viruses such as SARS-CoV2 [120].

In addition to missing some PRRs and associated signaling cascades, the absence of certain pro-inflammatory pathways may suggest *Aplysia* exhibits an inflammatory response different from vertebrates. A major component of the vertebrate inflammatory response is the formation of the NLRP3 inflammasome [121, 122]. While originally believed to have evolved in vertebrates, recent evidence suggests that the NLRP3 inflammasome may have antecedents in invertebrate innate immunity as orthologs of NLRP3 exhibit immune function in echinoderms and arthropods [123, 124]. The only *Aplysia* NLR identified does contain a Death-like domain, considered a possible predecessor to the CARD domain of NLRP3 in vertebrates, suggesting perhaps some conserved function [125]. However, the dearth of NLRs, lack of PYHIN proteins, and absence of RIPKs suggest that *Aplysia* does not generate inflammasomes in response to pro-inflammatory signaling [126].

Studies in bivalves suggest an ancestral molluscan innate immune system with a high degree of conservation with that of deuterostomes [3, 8, 127–129]. However, the absence of several PRRs and their adaptors suggests that the immune response in *Aplysia* may be substantially different not only from vertebrates but also from bivalves. Antiviral immunity in arthropods acts through different mechanisms than in vertebrates, notably lacking RLR antiviral signaling. Arthropods detect and respond to viruses via the RNA interference, TLR, JAK/STAT, and IMD/PGRP pathways [88, 130]. Perhaps heterobranchs gastropods, exhibiting a reduced immune complement compared to their bivalve relatives, have developed alternative, lineage specific immune pathways as occurred in arthropods.

## Conclusion

Together these results illustrate the difficulty of interpreting transcriptomic and genomics data in understudied lineages. Due to the overrepresentation of mammals and ecdysozoans in immune research, there is risk of erroneous interpretation of gene function based on similarity searches alone in mollusks. In these results, areas of broad conservation, notably in canonical toll-like receptor signaling were identified. However, *Aplysia* also exhibits areas of stark deviation from mammalian and even bivalve immunity, notably the absence of STING and MAVS. This illustrates the need for a healthy measure of caution when investigating immunity in *Aplysia*. By combining homology searches with annotation of conserved protein functional domains, an annotation set has been created that will provide a more nuanced basis for interpretation of immune-associated data in *Aplysia*. However, due to the in silico nature of this study, it is not possible to fully address potentially novel elements in *Aplysia* immunity. This dataset will serve as a springboard for future immune studies in *Aplysia* to further elucidate immune function in this venerable marine model.

## Methods

In order to identify a suite of potential immune associated genes in *Aplysia californica*, conserved protein domain annotation via InterProScan was combined with homology search and tree based ortholog identification via OrthoFinder.

InterProScan (IPS) is a software suite that annotates queried proteins via several algorithms for in silico functional characterization [131, 132]. Annotations are given not only for the InterPro database, but also participating databases such as PFAM, SMART, PANTHER. For this analysis, the publicly available AplCal3.0 RefSeq reference genome assembly (GCF_000002075.1), generated from whole genome shotgun sequencing by the Broad Institute, was used [133]. *Aplysia* RefSeq predicted protein models for this assembly (GCF_000002075.1_AplCal3.0_protein.faa) were annotated for conserved domains, motifs, and protein families via IPS (version 5.52–86.0) on the University of Miami Center for Computation Science PEGASUS supercomputer [132]. IPS config file used can be found in supplemental file SFile4. The resulting database was then searched for proteins annotated with conserved domain suites that characterize described immune-associated genes in other organisms. The list of genes of interest and their requisite domains was constructed by combining previous in silico immune gene studies in Pacific oyster *Crassostrea gigas* [3] and pond snail *Lymanaea stagnalis* [10]*.* This list was further expanded upon based on other reviews of immune-associated genes in vertebrates, mollusks, and arthropods [2, 4, 5, 9, 29–31, 33, 48, 55, 61, 63–65, 69, 84, 98, 110, 129, 134–141]. The full list of genes and requisite domains used can be found in supplemental file SFile5.

Conserved domain family screening of a proteome can recover putative members of known gene families with high confidence. Nevertheless, for proteins that are difficult to distinguish from a domain level alone (ie IkB with only Ankyrin repeat domains), this approach alone is insufficient. Furthermore, in several protein families of interest, molluscan proteins lack several domains or contain extra domains relative to their vertebrate and arthropod orthologs. In order to avoid missing these genes, previously identified molluscan representatives of these gene families were used in homology-based searches and validated with conserved domain data.

For more traditional homology searches for immune genes, reference predicted protein models for *Aplysia*, *Crassostrea gigas* (GCA_000297895.1_oyster_v9_protein.faa)*,* and *Biomphalaria glabrata* (GCF_000457365.1_ASM45736v1_protein_Bglabrata.faa) from the RefSeq database [142, 143], and reference proteomes for *Homo sapiens* (UP000005640_9606_Homo_Sapies) and *Drosophila melanogaster* (UP000000803_7227_DroMel) from UNIPROT were analyzed with OrthoFinder. OrthoFinder combines reciprocal, all-against-all BLAST homology search with hierarchical clustering to identify probable orthologs and clusters of orthologs called orthogroups [144]. These collections of orthologs were then queried for genes identified as immune associated in *C. gigas* [3]*, B glabrata* [5], human, and *Drosophila* (SFile2).

The two sets of putative immune genes generated by these two analyses were then inspected and compared by hand to verify that hits contained requisite functional domains and were likely relatives of target genes. Data manipulation was done in the R statistical environment using RStudio and the tidyverse software suite [145, 146].

TIR domains of the longest protein isoform of each putative *Aplysia* TLR gene were extracted from RefSeq predicted protein fasta files using predicted TIR domains from IPS using tidyverse and the Biostrings R package [147]. Extracted TIR domain sequences were aligned using the msa R package with default clustal parameters [148]. A neighbor joining tree from Aplysia TLR TIR multi-sequence alignments was constructed with the Phangorn R package using the standard scripts for amino acid trees from the developer [149, 150]. Briefly, tree was built with a k of 4, a proportion of invariable sites of 2, and using the Jones-Taylor-Thornton model and bootstrapped with 1000 iterations. The resulting tree was visualized with the treeio and ggtree R packages [151–154].

TLR proteins for phylogenetic tree of TLRs were extracted from publicly available InterPro annotations of proteins from the UniprotKB data base for *Biomphalaria glabrata* (Bglab, UP000076420_IPS), *Crassosteraea gigas* (Cgig, UP000005408), *Nematostella vectensis* (Nvect, UP000001593), *Drosophila melanogaster* (Dromel, UP000000803), *Strongylocentrotus purpuratus* (Spurp, UP000007110)*,* and human (Hs, UP000005640). These files were queried for TLRs based on TIR domain and LRR domains as was done with AplCal3.0 IPS annotation described above. Predicted TLR sequences were download from the UnirpotKB (or RefSeq for *Aplysia* and *Nematostella*) and used to generate a tree as described above for TIR domain sequences. The tree was not bootstrapped due to long iteration time. Tree was imported and visualized with treeio and ggtree.

## Supplementary Information

**Additional file 1: Supplemental File SFile1.** Full results of InterProScan annotation of the *Aplysia* reference proteome (AplCal3.0 GCF_000002075.1).

**Additional file 2: Supplemental File SFile2.** OrthoFinder Results. OrthoFinder Results. First page contains the overall statistics from OrthoFinder about orthogroups. The second page contains pairwise ortholog counts among species surveyed. Page three contains per species statistics. The remaining pages contain orthologs and ortholog families (orthogroups) identified by OrthoFinder using reference proteomes for Aplysia, *Crassostrea gigas* (GCA_000297895.1_oyster_v9_protein.faa), and *Biomphalaria glabrata* (GCF_000457365.1_ASM45736v1_protein_Bglabrata.faa) from the RefSeq database, and reference proteomes for *Homo sapiens* (UP000005640_9606_Homo_Sapies) and *Drosophila melanogaster* (UP000000803_7227_DroMel). Each page labeled is labeled with the accession number and organism name for each organism. The last page is a complete collection of all proteins assigned to all computed orthogroups. The complete output of OrthoFinder including gene trees are available from the author upon request.

**Additional file 3: Supplemental File SFile3.** Putative *Aplysia* Immunome gene set. A Complete list of RefSeq gene and protein identifiers of genes identified as putative immune associated gene based on InterProScan and OrthoFinder results.

**Additional file 4: Supplemental File SFile4.** InterProScan configuration file. This file contains the configuration parameters used to annotate the Aplysia AplCal3.0 reference proteome.

**Additional file 5: Supplemental File SFile5.** Target genes and requisite InterPro domains. The list of genes of interest and their requisite domains used to identify putative immune genes in *Aplysia californica*. The list combines data from previous mollusk immunome in silico studies in Pacific oyster *Crassostrea gigas* [3] and pond snail *Lymanaea stagnalis* [10] and reviews of immune-associated genes in vertebrates, mollusks, and arthropods [2, 4, 5, 9, 29–31, 33, 47, 54, 60, 62–64, 68, 88, 98, 117, 121–129]. Genes are categorized according to their broad immune function and more specific gene family. Required InterPro domains are listed as strict (as appears in human) or relaxed (required domains as seen in non-human or non-mammalian systems). Human UNIPROT identifiers of example genes are listed and were futher used in OrthFinder homology search comparisons. For some genes that exist only in ecdysozoans, the *Drosophila* UNIPROT identifier is also given. The DOI and link to the source literature is also provided.

## Data Availability

All data generated or analyzed during this study are included in this published article and its supplementary information files. All scripts used in this analysis are available on GitHub [https://github.com/Nicholas-Kron/Aplysia_Immunome].

## Abbreviations

A2M: Alpha-2-macroglobulin containing proteins
ABCF1: ATP-binding cassette sub-family F member-like
ADAM: A disintegrin and metalloproteinase
ADAR: Double-stranded RNA-specific adenosine deaminase
ADH: Alcohol dehydrogenase-like
AGO: Argonaute
AIF: Mitochondrial apoptosis-inducing factor
AIM2-like/IFI16-like: Pyrin and hematopoietic interferon-inducible nuclear (HIN) domain (PYHIN) proteins
AP-1: Activating protein 1 family
APOBEC3: Apolipoprotein B Editing Complex 3 proteins
ARD1: E3 ubiquitin-protein ligase TRIM23
ARM-TIR: Armadillo fold and TIR domain containing proteins
ASK: Apoptosis signal-regulating kinase (MAP3Ks)
Bcl2-like: Apoptosis regulator Bcl2- like
BST2L: Tetherines (Bone marrow stromal antigen 2-like)
C1qDC: C1q domain containing proteins
C3/4/5: Complement proteins 3/4/5
CARD: Caspase recruitment domain
CAT: Catalase
CAV: Caveolin
CD109: Cluster of Differentiation 109
CD14: Monocyte differentiation antigen CD14
CD36-like: CD36/scavenger receptor class B member 1-like
CFB: Factor B-like
cGAS: Cyclic GMP-AMP synthase
COR: C-terminal of Roc domain
CRADD: Caspase and RIP adapter with death domain
CRD: Carbohydrate recognition domain
CREP: VIgL _C-type lectin-related protein
CTD: c-terminal domain
CTLDC: C-type lectin domain containing proteins
CUL: Cullin
DC: Domain containing
DD: Death Domain
DDIT3/CHOP: DNA damage-inducible transcript 3 protein (CHOP, c/EBPz/GADD153)
DDX_DEAD_Q: ATP-dependent RNA helicase with DEAD Q motif
DDX1: ATP-dependent RNA helicase DDX1
DDX21: ATP-dependent RNA helicase DDX21
DDX41: ATP-dependent RNA helicase DDX41
DExD/H-box: DExD/H-box helicases - other
DHX_DUF1605: ATP-dependent RNA helicases with Domain of unknown function 1605
DHX15: ATP-dependent RNA helicase DHX15
DHX9: ATP-dependent RNA helicase DHX9
DLD: Death-like Domain
DNAPK: DNA-dependent protein kinase-like
DUOX: Dual oxidase
DUOXA: Dual oxidase maturation factor
ECSIT: Evolutionarily conserved signaling intermediate in Toll pathway
EEP: Exonuclease-Endonuclease-Phosphatase
EGF-TIR: Epidermal growth factor and TIR domain containing proteins
ERK1/2: Extracellularly Regulated Kinase 1/2
ERK3/4: Extracellularly Regulated Kinase 3/4
ERR: Estrogen receptor/oestrogen-related receptors
ETX/MTX-2: Epsilon toxin
FADD: FAS-associated death domain protein
FAIM: Fas apoptotic inhibitory molecule
FBGDC: Fibrinogen domain containing protein
FBXL: F-box/LRR-repeat protein
FREP: VIgL_fibrinogen-related proteins
FYN: Tyrosine-protein kinase Fyn
Gilt: Gilt
GIMAP: GTPase IMAP family member
GNBP: Gram-negative bacteria binding-protein
GPX: Glutathione peroxidase
GREP: VIgL _Galectin-related protein
GRP: Beta 1,3-glucan recognition proteins
GRX: Glutaredoxin
GSR: Glutathione reductase
GST: Glutathion S transferase
H-type lectin: H-type lectins
HMGBs: High mobility group box proteins
HOIL1: RanBP-type and C3HC4-type zinc finger-containing protein 1 RBCK1
HOIP: E3 ubiquitin-protein ligase RNF31
HSF: Heat shock factor protein
HSP20: Heat shock 20 kDa protein
HSP70: Heat shock 70 kDa protein
HSP90: Heat shock 90 kDa protein
HTRA2: Serine protease HTRA2
I-type lectins: Immunoglobulin lectins
IAPs: Baculoviral IAP repeat-containing proteins
IFI44: Interferon-induced protein 44
IFITML: Interferon-induced transmembrane protein-like
IFN: Interferon
IFNAR: Interferon alpha/beta receptor
IG: Immunoglobulin
IG-TIR: Imunoglobulin and TIR domain containing proteins
IkB: Inhibitor of NFkB
IKK: Inhibitor of nuclear factor kappa-B kinase subunit alpha
IKKe/i: Inhibitor of nuclear factor kappa-B kinase subunit epsilon
IKKg/NEMO: NF-kappa-B essential modulator
IL-17: Interleukin 17
IL17R: Interleukin 17 receptor
IPS: InterProScan
IRAK: Interleukin-1 receptor-associated kinase 1
IRF: Interferon regulatory factor
ISG: Interferon stimulated gene
JAK: Tyrosine-protein kinase JAK
JNK: Mitogen-activated protein kinase 8
JUN: Activating protein 1 JUN
Ku70/80: ATP-dependent DNA helicase II/X-ray repair cross-complementing protein 5/6
L-type lectin: Legume-like lectin
LAAO: L-Amino Oxidase
LBP/BPI: Lipopolysaccharide-binding protein/Bactericidal/permeability-increasing protein
LITAF: Lipopolysaccharide-induced tumor necrosis factor-alpha factor
LPS: Bacterial liposaccharide
LRR: Leucine Rich Repeat
LRR-IG: Leucine rich repeate and immunoglobin domain containing protein
LRR-other: Leucine rich repeat containg protein
LRRFIP1: Leucine-rich repeat flightless-interacting protein 1-like
LUBAC: Linear ubiquitin chain assembly complex
MAC: Membrane attack complex
MACPF: MACPF domain containing protein (Perfoin-like)
MAP2K5: Mitogen-activated protein kinase kinase 5 (MEK5)
MAP2Ks: Dual specificity mitogen-activated protein kinase kinase
MAP3K1/2: Mitogen activated protein kinase kinase kinase 2/3
MAP3K10: Mitogen activated protein kinase kinase kinase 10 (MST, MLK2)
MAP3K12/13: Mitogen activated protein kinase kinase kinase 12/13 (DLK, MEKK12/13)
MAP3K4: Mitogen activated protein kinase kinase kinase 4 (MEKK4, MTK1)
MAP3Ks: Dual specificity mitogen-activated protein kinase kinase kinase
MAPKs: Mitogen activated protein kinases
MAVS: Mitochondrial Antiviral-Signaling Protein
MBP: Mannose-binding lectin
MD2: Myeloid Differentiation factor 2/Lymphocyte antigen 96
MEGF10: Multiple epidermal growth factor-like domains protein 10
MEX3C: RNA-binding E3 ubiquitin-protein ligase MEX3C
MIF: Macrophage migration inhibitory factor
MK2: MAP kinase-activated protein kinase 2
MKP: Mitogen activated protein kinase phosphatase
MnSOD: Superoxide Dismutase (Mn/Fe)
MRE11: Double-strand break repair protein MRE11-like
MVP: Major Vault Protein
Mx: Interferon-induced GTP-binding protein Mx
MyD88: Myeloid differentiation primary response protein
NACHT: NAIP, CIITA, HET-E and TP1 NTPase domain
NAP1: Nck-associated_protein-1
NFkB: Nuclear factor kB
NLR: NOD-like receptor
NOS: Nitric-oxide synthase
NOX: NADPH oxidase
NXN: Nucleoredoxin
OAS: 2′-5′-oligoadenylate synthetase
OrTIR: Orphan TIR proteins
P-type lectin: Mannose 6-phosphate receptor homology domain containing
p38: p38 MAPK/ MAPK14
PAMP: Pathogen associated molecular pattern
PARPs: Protein mono-ADP-ribosyltransferase
PFP: Pore forming protein
PGRP: Peptidoglycan recognition proteins
PIWI: Piwi-like
PKR: EIF2AK2/PKR-like
PP1: Serine/threonine-protein phosphatase PP1
PRDX: Peroxiredoxin
proPO: Phenoloxidase
PRR: Pattern recognition receptor.
PRX: Peroxidases
PRXDN: Peroxidasin
PYHIN: Pyrin and hematopoietic interferon-inducible nuclear (HIN) domain
RAD50: DNA repair protein RAD50
RanBP2DC: RanBP2 domain containing proteins
RIGI/IPS1_CARD_DC: RIG-I and MAVS type CARD domain containing proteins
RIOK3: RIO Kinase 3
RIPKs: Receptor-interacting serine/threonine-protein kinase
RLR: RIG-I-like receptors
RLR: RIG-I-like receptor
RNApol_III: DNA-directed RNA polymerase III
RNASEL: Ribonuclease L
RNF134/RIPLET: E3 ubiquitin-protein ligase RNF135/RING finger protein leading to RIG-I activation
ROC: Ras of complex domain
ROCO: ROC and COR domain
RSADs: Viperins (Radical S-adenosyl methionine domain-containing proteins)
RXR: Retinoid X receptor
SAMHD: Deoxynucleoside triphosphate triphosphohydrolase SAMHD
SARM: NAD(+) hydrolase SARM1
SCF: SKIP1, Cullin, F-box protein complex
SIAE: Sialate O-acetylesterase
SKIP1: S-phase kinase-associated protein 1
SMAD: “Small” worm phenotype and “Mothers Against Decapentaplegic” homology domain
SOCS: Suppressor of cytokine signaling
SOD: Superoxide Dismutase (Cu/Zn)
SRCR: Scavenger receptor cysteine-rich domain containing protein
SRCR: Receptor Cysteine-Rich (SRCR) Domain
STAT: Signal transducer and activator of transcription
STING/MITA: Stimulator of interferon genes
TAB1: TGF-beta-activated kinase 1 and MAP3K7-binding protein 1
TAB2/3: TGF-beta-activated kinase 1 and MAP3K7-binding protein 2/3
TAK1: MAP3K7/TGF-beta-activated kinase 1
TANK: TRAF family member-associated NF-kappa-B activator
TBK1/NAK: TANK-binding kinase 1/ NFkB activated Kinase
TBKBP1/SINTBAD: TANK-binding kinase 1-binding protein 1
TEP: Thioester containing protein (thioester)
TIR: Toll/interleukin-1 receptor homology (TIR) domain
TIR-TPR: TIR and tetratrico peptide repeat containing proteins
TIRAP/Mal: Toll/interleukin-1 receptor domain-containing adapter protein
TIRDC: TIR domain containing
TLR: Toll-like receptor
TNAP3/A20: Tumor necrosis factor alpha-induced protein 3/A20-like
TNF: Tumor necrosis factor
TNFR: Tumore necrosis factor receptor
TOLLIP: Toll-interacting protein
TPL2/MAP3K8: Mitogen-activated protein kinase kinase kinase 8
TRADD: Tumor necrosis factor receptor type 1-associated DEATH domain protein
TRAF: TNF receptor-associated factors
TRAM/TICAM2: TIR domain-containing adapter molecule 2
TRBP: RISC-loading complex subunit TARBP
TRIF/TICAM1: TIR domain-containing adapter molecule 1
TRIM: E3 ubiquitin-protein ligase TRIM
TRIM25/65: E3 ubiquitin/ISG15 ligase TRIM25/65
TRIM4: E3 ubiquitin-protein ligase TRIM31
TRIM5: Tripartite motif-containing protein 5
TRXDC: Thioredoxin domain-containing protein
VIgL: Variable Immunoglobulin and Lectin domain containing molecules
ZAP: Zinc-finger antiviral proteins
ZBP/DAI: Z-DNA-binding proteins
ZNFX1: Zinc finger NFX1-type containing 1.
